# Measuring dissolved black carbon in water via aqueous, inorganic, high-performance liquid chromatography of benzenepolycarboxylic acid (BPCA) molecular markers

**DOI:** 10.1371/journal.pone.0268059

**Published:** 2022-05-26

**Authors:** Riley Barton, Sasha Wagner

**Affiliations:** Department of Earth and Environmental Sciences, Rensselaer Polytechnic Institute, Troy, New York, United States of America; University of Maryland Center for Environmental Science, UNITED STATES

## Abstract

Dissolved black carbon (DBC) is the condensed aromatic portion of dissolved organic matter produced from the incomplete combustion of biomass and other thermogenic processes. DBC quantification facilitates the examination of the production, accumulation, cycling, transformation, and effects of biologically recalcitrant condensed aromatic carbon in aquatic environments. Due to the heterogeneous nature of DBC molecules, concentrations are difficult to measure directly. Here, the method for DBC quantification consists of oxidizing condensed aromatic carbon to benzenepolycarboxylic acids (BPCAs), which are used as proxies for the assessment of DBC in the original sample. The concentrations of oxidation products (BPCAs) are quantified using high-performance liquid chromatography. DBC concentrations are determined from the concentration of BPCAs using a previously established conversion factor. Details and full descriptions of the preparative and analytical procedures and techniques of the BPCA method are usually omitted for brevity in published method sections and method-specific papers. With this step-by-step protocol, we aim to clarify the steps of DBC analysis, especially for those adopting or conducting the BPCA method for the first time.

## Introduction

The heating and partial combustion of organic matter (e.g., during wildfires and the burning of fossil fuels) fundamentally alters the molecular structure of unburned biomass [1]. Pyrogenic and other thermogenic residues are best described as a continuum of organic compounds that range from mildly altered, biolabile molecules to highly altered, biorefractory, condensed aromatic compounds that are typically formed at high charring temperatures [2, 3]. The condensed aromatic portion of partially combusted organic matter is termed “black carbon” (BC; [3]). Due to its condensed aromatic molecular structure, BC is highly resistant to biodegradation and is thus stabilized in soils for centuries to millennia [4, 5]. The ubiquity and long-term storage of BC in soils suggest that the production of BC during wildfires may serve to partially offset fire-derived carbon emissions [6]. BC is an important component of the global carbon cycle in terms of carbon sequestration and therefore draws research interest from a wide range of disciplines, such as biogeochemistry, climatology, oceanography, and soil science.

Research gaps that currently prevent sufficient representation of BC cycling in global models include poorly understood fluxes between major and intermediate carbon reservoirs and biogeochemical processes that control the fate of BC in the environment at local and regional scales. Some portion of the BC deposited to soils and sediments “leaks out” over time, entering rivers in the form of dissolved BC (DBC). An estimated 18–27 Tg of DBC is exported globally by rivers each year, which may reduce the presumed efficacy of long-term BC storage on land [7, 8]. The mobilization of DBC serves as a link between terrestrial and marine reservoirs and DBC concentrations are usually higher in rivers (1.5 to 140 uM-C) than in the ocean (0.08 to 2.5 uM-C; [3]). Leaching experiments indicate a high degree of variability in terms of the amount and composition of DBC leached from soils, charcoal, and pyrogenic aerosols [9–13]. Due to the heterogeneous nature of DBC compounds, which exist in an even more complex dissolved organic matter matrix, the direct quantification of these condensed aromatic molecules is analytically challenging. The benzenepolycarboxylic acid (BPCA) method is one of the most widely utilized methods for condensed aromatic DBC quantification in aquatic environments (see Wagner et al. [3, 14] for further discussion on this) and is the analytical protocol we describe here.

**Fig 1:**
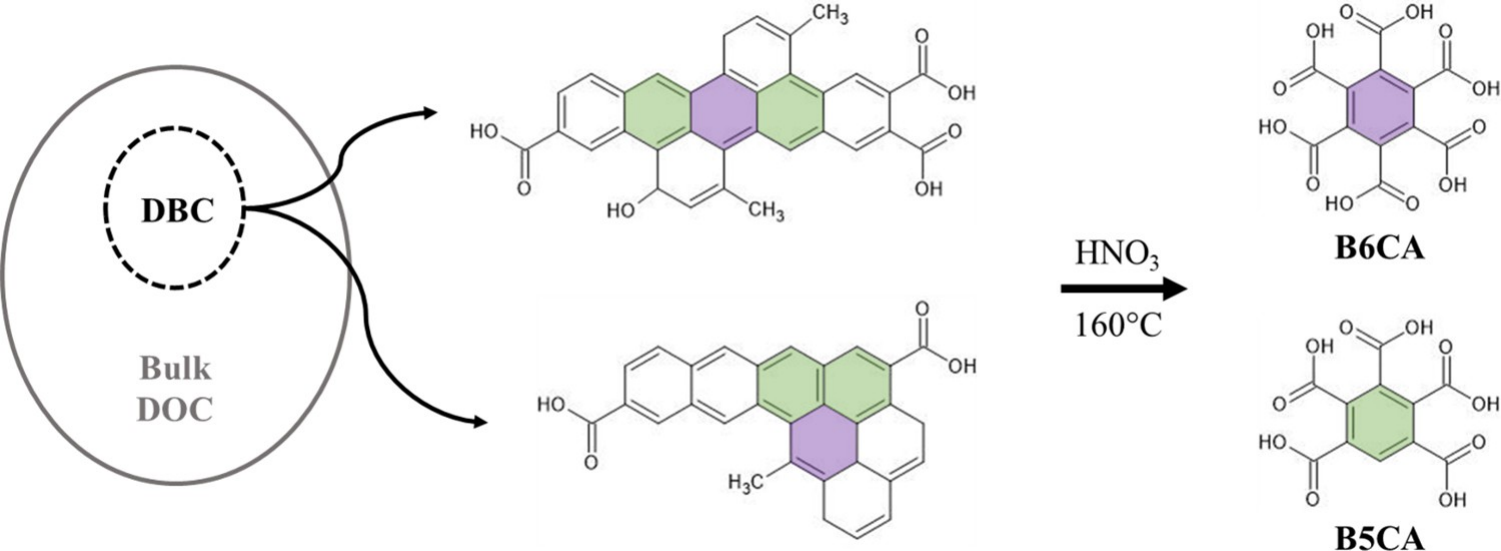
Oxidation of DBC to BPCAs. Dissolved black carbon (DBC) makes up a portion of bulk dissolved organic carbon (DOC). The two examples of possible DBC molecular structures shown were proposed by Dittmar and Paeng [24] based on ultrahigh-resolution mass spectral analysis of ocean water by Dittmar and Koch [25]. When black carbon molecules are oxidized with nitric acid and heat, they produce benzenepolycarboxylic acids (BPCAs). Oxidation products from highly condensed aromatic structures would include highly substituted BPCAs; benzenehexacarboxylic acid (B6CA) and benzenepentacarboxylic acid (B5CA). Structural regions of the two exemplary DBC molecules are colored purple and green to indicate how the condensed aromatic core could oxidize to produce either B6CA or B5CA [16].

The BPCA method was first established for quantifying BC in soils [15] and has since been adapted for measuring DBC in water [16, 17]. While the BPCA method was originally developed for the study of pyrogenic condensed aromatic carbon, its use has since expanded to encompass non-pyrogenic condensed aromatic carbon classes such as those present in crude oil and asphaltene fractions [18, 19]. Thus, we define DBC as the condensed aromatic portion of bulk dissolved organic carbon (DOC) that passes through a filter (usually 0.1 to 0.7 um pore size) and is detectable as BPCA molecular markers upon nitric acid oxidation (Fig 1). The oxidation of condensed aromatic DBC molecules yields a mixture of substituted BPCAs, including benzenetricarboxylic acids (B3CAs), benzenetetracarboxylic acids (B4CAs), benzenepentacarboxylic acid (B5CA), and benzenehexacarboxylic acid (B6CA) as well as nitrated BPCAs [16, 20]. It is important to note here that the BPCA method detects molecular oxidation products (not native DBC structures), which represent only a fraction of all condensed aromatic carbon present in the original sample. This fraction was originally estimated to be ~30% on a per-carbon basis for DBC; determined from the average amount of carbon retained from the oxidation of polycyclic aromatic hydrocarbon standards and the average molecular formula of marine condensed aromatic structures inferred from ultrahigh-resolution mass spectroscopy [16, 20]. Thus, we rely on conversion factors to estimate DBC concentrations from the concentrations of measured BPCAs. The conversion factors utilized by the aquatic black carbon community do vary depending on the chromatographic method employed, specific BPCAs quantified, and/or DBC standard material used [16, 20–23].

The oxidation of non-pyrogenic samples has been shown to produce the less-substituted BPCAs (B3CAs and B4CAs) potentially through carbonization reactions during oxidation of large amounts of organic carbon (> 5mg-OC per 2 mL of nitric acid) [26]. Therefore, the protocol we describe here limits the amount of OC oxidized and utilizes an equation derived from the power relationship between established DBC concentrations and the sum of B5CA and B6CA only [21]. Current conversion factors to translate BPCA to DBC concentrations, including the one we employ here, are based upon the oxidation patterns of polyaromatic hydrocarbon standards [20] and ultrahigh-resolution mass spectral data [16]. Other conversion factors have been developed from the oxidation of graphene oxide [23]. However, none of these condensed aromatic carbon standards or analytical approaches is capable of representing the entire suite of DBC molecules that exist in aquatic matrices. Therefore, we recognize that the relationship (the conversion factor) between the DBC compound class and its BPCA oxidation products may be altered or further refined with continued research. Therefore, we strongly encourage the publication of raw BPCA concentrations alongside calculated DBC concentrations whenever the BPCA method described here is used to produce the data. In doing so, works involving published DBC concentrations can be easily adapted if new conversion factors are established and will facilitate the synthesis and scaling of DBC data among studies. Should it be shown that non-condensed aromatic structures can yield considerable BPCA products upon oxidation, the definition of DBC and its context in global biogeochemical cycling would need to be revisited. To assist in answering these questions, we provide a detailed protocol for those looking to adopt a method for the quantification of this biogeochemically relevant, highly photolabile, yet environmentally long-lived fraction of DOC.

The protocol we use to quantify DBC in aquatic samples via BPCA analysis is summarized here. DOC is isolated from the filtered water sample via solid-phase extraction (SPE) following Dittmar et al. [27]. The adsorbed DOC is eluted from the SPE cartridges using methanol and recovered as solid-phase extracted DOC (SPE-DOC). A volume of methanol eluate equating to ~0.5 mg-DOC is placed in a glass ampule and evaporated. Concentrated nitric acid is added to the ampule containing the DOC eluate residue and is flame-sealed. The sealed ampules are oxidized in an oven for 6 hours at 160°C [28]. After oxidation, the ampules are opened and the reaction mixture is evaporated to dryness under a stream of nitrogen gas. The remaining BPCA-containing residue is re-dissolved in a phosphoric acid solution for subsequent analysis. The BPCAs are separated using an established high-performance liquid chromatography (HPLC) method and quantified using a diode array detector. This method separates B6CA and B5CA peaks, which are identified by comparing retention times to that of BPCA standards. Sample BPCA concentrations are determined by external calibration and are scaled to DBC concentrations using the Stubbins et al. [21] conversion factor.

The BPCA method described in this protocol is not new and details of method development are published elsewhere [17]. However, the many small, yet important, details and full descriptions of the preparative and analytical techniques are usually omitted for brevity in published method sections and method-specific papers. These technical omissions make it difficult for researchers who are looking to adopt or refine the BPCA method. With this detailed laboratory protocol, we outline the step-by-step considerations and techniques omitted from previous publications. We aim to lower the barrier to entry for those who want to incorporate BPCA analysis and DBC quantification into their research but do not have a high level of access to those familiar with the method. The contribution of additional perspectives and studies examining DBC will further the understanding of the production, fate, and impact of DBC in the environment.

## Materials and methods

The protocol described in this peer-reviewed article is published on protocols.io, **https://dx.doi.org/10.17504/protocols.io.5qpvoy2b9g4o/v2**, and is included for printing as S1 File with this article.

### Expected results

**Fig 2:**
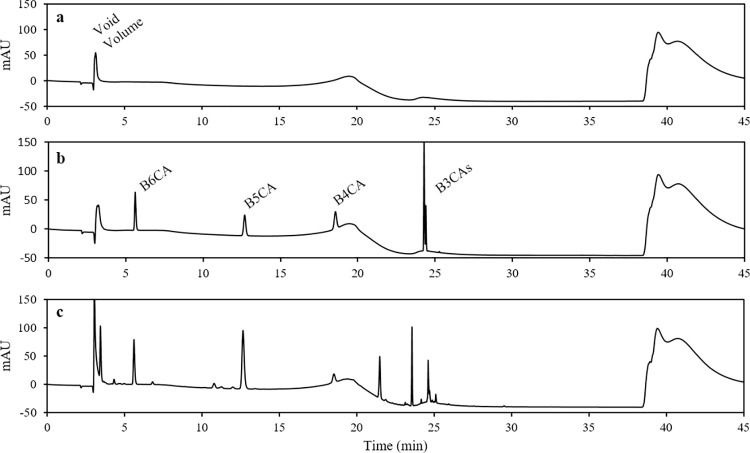
HPLC chromatograms of (a) mobile phase gradient as well as benzenepolycarboxylic acid separation in (b) a standard mixture and (c) a dissolved organic matter sample from a fire-affected headwater stream. As seen in each chromatogram, the initial peak is representative of void volume and the baseline variation is due to changes in mobile phase gradient. The BPCA standard peaks in **b** are indicative of B6CA, B5CA, a B4CA (1,2,4,5-B4CA), and three B3CAs (1,2,4-B3CA, 1,2,3-B3CA, 1,3,5-B3CA). B3CAs and B4CA are not quantified as part of this method but are included in chromatographic analyses to confirm complete elution of sample.

By following this protocol, you will obtain concentrations of B6CA and B5CA produced from the oxidation of DBC in water samples. Sample BPCA peaks are identified using absorbance spectra and/or peak retention times of BPCA standards. Chromatograms of BPCA standards should not contain extraneous peaks (besides void volume) but may have baseline variations resulting from mobile phase gradients (Fig 2A and 2B). Sample chromatograms will likely have many extraneous peaks that reflect oxidation products other than B5CA and B6CA, however, targeted BPCAs are usually baseline-separated (Fig 2C). DBC concentrations (in uM-C) are calculated from B5CA and B6CA concentrations (in nM-BPCA) using the following equation (from Stubbins et al. [21]): [DBC] = 0.0891 x ([B6CA] + [B5CA])^0.9175^

Using the described protocol, we have measured and published DBC data for diverse aquatic systems, including major and minor rivers, along oceanic depth profiles, throughfall and stemflow, petroleum-contaminated groundwater, and coastal environments [13, 18, 29–32]. Although DOC analysis is not explicitly described in the current protocol, we strongly recommend that users of this method measure and share bulk DOC concentrations alongside BPCA and DBC concentrations. Ratios of DBC to DOC are often reported because they can vary between fresh and marine water, vary between types of rivers, and decrease with increased photodegradation [3, 31, 33]. Molar ratios of B6CA to B5CA are also useful as a proxy to express the degree of relative condensed aromaticity of DBC among different samples. Higher B6CA:B5CA ratios indicate DBC pools containing larger fused aromatic ring structures [34]. B6CA to B5CA ratios have been found to decrease with increased photodegradation, are usually lower in oceans compared to rivers, and have been found to be higher at high river flow than low river flow [21, 31, 33].

Here, we display a subset of DBC data originally reported by Wagner et al. [31] that was determined using this protocol. The dataset includes both oceanic and riverine samples to show examples of distinctive DBC results that may be observed for diverse aquatic systems. Considering the source and prior biogeochemical processing of a target water sample is critical for making any necessary sample volume adjustments or other accommodations during sample preparation and analysis. For example, oceanic samples have significantly lower SPE efficiencies (i.e. lower DOC recovery by SPE), lower DOC and DBC concentrations, and lower DBC:DOC and B6CA:B5CA ratios than riverine samples (Table 1). Since proportions of DBC:DOC and B6CA:B5CA are inherent for any given sample, we would conclude that higher sample volumes are needed for oceanic samples than river samples to recover sufficient amounts of DBC for robust BPCA detection and quantification. When working with new samples, where DBC:DOC and B6CA:B5CA ratios are truly unknown, we recommend oxidizing 0.5 mg SPE-DOC to gain a preliminary sense of sample composition with regards to condensed aromatic DBC. It is important to remember that, since B6CA:B5CA ratios are typically < 1 (Table 1), minimal sample volumes required for DBC analysis should be estimated from the expected yield of B6CA. The limits of detection and quantification for the BPCA method using the protocol described here are 1.5 and 5 uM-B6CA, respectively. Based upon these analytical detection limits and our prior experience [17], we recommend a lower reporting limit of 20 uM-B6CA (in vial, to be injected for HPLC separation). Oftentimes, BPCA ratios such as B6CA:B5CA are used to describe relative degree of DBC aromaticity and to trace changes in DBC sources and biogeochemistry over time [21, 34]. As B6CA is almost always present in lower concentrations than B5CA in aqueous samples, establishing a lower reporting limit of 20 uM-B6CA ensures the calculation of robust DBC concentrations and BPCA ratios. Furthermore, if users adhere to these suggested limits, then samples prepared following this protocol could also be used for BPCA-specific stable carbon isotopic analysis [17].

**Table 1:** A subset of DBC data originally published by Wagner et al. [3,1].

Site	SPE-DOC Recovery	DOC (μM-C)	DBC (μM-C)	DBC: DOC (%)	B6CA (nM)	B5CA (nM)	B6CA: B5CA
Pacific Ocean	40%	81	0.62	0.8	1.6	6.7	0.23
Atlantic Ocean	48%	63	0.78	1.2	2.1	8.6	0.24
Amazon River	78%	331	25	7.6	140	332	0.42
Congo River	64%	540	37	6.8	234	473	0.49
Northern Dvina River	64%	519	42	8.0	295	516	0.57
Kolyma River	70%	327	20	6.2	112	260	0.43
Mississippi River	61%	247	18	7.3	75	251	0.30

Water samples were collected from the Pacific and Atlantic Oceans as well as the Amazon, Congo, Northern Dvina, and Mississippi Rivers. Each sample was analyzed for dissolved organic carbon (DOC), recovery of dissolved organic carbon after solid-phase extraction (SPE-DOC), dissolved black carbon (DBC), the ratio of DBC to DOC, the concentrations of B6CA and B5CA oxidation products, and the ratio of B6CA to B5CA.

However, reaching this target B6CA concentration requires different sample volumes for biogeochemically distinct sample types, like river and open ocean water. If we assume a typical river sample has a DOC concentration of 520 uM, DBC:DOC ratio of 8%, an extraction efficiency of 65%, and a B6CA:B5CA ratio of 0.57, then a minimum of 20 mL of sample volume is required to recover sufficient carbon for DBC analysis. In contrast, if we assume a typical open ocean sample has a DOC concentration of 50 uM, DBC:DOC ratio of 1% and an extraction efficiency of 45%, and a B6CA:B5CA ratio of 0.23, then a minimum of 4.5 L of sample volume would be necessary. These estimates are conservative, to ensure robust BPCA measurement. Required sample volumes may be further reduced depending upon sample composition, extraction efficiency, injection volumes, specific detection limits of the instrument used for separation and detection, etc. Users of this method are encouraged to use the BPCA preparation and calculation spreadsheets (S2 File) to estimate sample volumes needed to conduct robust DBC analyses in the specific aquatic system under study.

## Supporting information

S1 File Protocol collection. Step-by-step protocol, also available on protocols.io.
(PDF)

S2 File Example PPL-BPCA calculations spreadsheet, also available on protocols.io. (XLSX)
