# Supplementary material for: Measuring dissolved black carbon in water via aqueous, inorganic, high-performance liquid chromatography of benzenepolycarboxylic acid (BPCA) molecular markers
Source: PLoS One. 2022 May 26;17(5):e0268059. doi: 10.1371/journal.pone.0268059 (PMC9135288; doi:10.1371/journal.pone.0268059)
Supplement: S1 File — Step-by-step protocol, also available on protocols.io. (PDF) [file pone.0268059.s001.pdf]

OPEN ACCESS

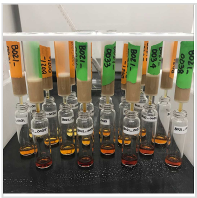

2

May 10, 2022

Measuring dissolved black carbon in water via aqueous, inorganic, high performance liquid chromatography of benzenepolycarboxylic acid (BPCA) molecular markers V.2

Riley Barton[1], Sasha Wagner[1]

[1]Department of Earth and Environmental Sciences, Rensselaer Polytechnic Institute, Troy, New York, US A

1

dx.doi.org/10.17504/protocols.io.5qpvoy2b9g4o/v2

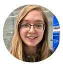

Riley Barton
Department of Earth and Environmental Sciences, Rensselaer P...

Dissolved black carbon (DBC) is the condensed aromatic portion of dissolved organic matter produced from the incomplete combustion of biomass and other thermogenic processes. DBC quantification facilitates the examination of the production, accumulation, cycling, transformation, and effects of biologically recalcitrant condensed aromatic carbon in aquatic environments. Due to the heterogeneous nature of DBC molecules, concentrations are difficult to measure directly. Here, the method for DBC quantification consists of oxidizing condensed aromatic carbon to benzenepolycarboxylic acids (BPCAs), which are used as proxies for the assessment of DBC in the original sample. The concentrations of oxidation products (BPCAs) are quantified using high-performance liquid chromatography. DBC concentrations are determined from the concentration of BPCAs using a previously established conversion factor. Details and full descriptions of the preparative and analytical procedures and techniques of the BPCA method are usually omitted for brevity in published method sections and method-specific papers. With this step-by-step protocol, we aim to clarify the steps of DBC analysis, especially for those adopting or conducting the BPCA method for the first time.

DBC Quantification Materials List.xlsx

Example PPL-BPCA Calculations.xlsx

DOI

dx.doi.org/10.17504/protocols.io.5qpvoy2b9g4o/v2

Citation: Riley Barton, Sasha Wagner Measuring dissolved black carbon in water via aqueous, inorganic, high performance liquid chromatography of benzenepolycarboxylic acid (BPCA) molecular markers https://dx.doi.org/10.17504/protocols.io.5qpvoy2b9g4o/v2

This is an open access protocol distributed under the terms of the **Creative Commons Attribution License** (https://creativecommons.org/licenses/by/4.0/), which permits unrestricted use, distribution, and reproduction in any medium,

Riley Barton, Sasha Wagner 2022. Measuring dissolved black carbon in water via aqueous, inorganic, high performance liquid chromatography of benzenepolycarboxylic acid (BPCA) molecular markers. **protocols.io**
https://protocols.io/view/measuring-dissolved-black-carbon-in-water-via-aque-b86arzae
Riley Barton

Additional details were added to the protocol after peer-review. Minor updates were made to the calculations spreadsheet.

dissolved black carbon, high performance liquid chromatography, benzenepolycarboxylic acid, aqueous separation, pyrogenic carbon, condensed aromatic carbon, biogeochemistry, organic geochemistry, aquatic chemistry

protocol ,

May 10, 2022

May 10, 2022

62370

Citation: Riley Barton, Sasha Wagner Measuring dissolved black carbon in water via aqueous, inorganic, high performance liquid chromatography of benzenepolycarboxylic acid (BPCA) molecular markers https://dx.doi.org/10.17504/protocols.io.5qpvoy2b9g4o/v2

This is an open access protocol distributed under the terms of the **Creative Commons Attribution License** (https://creativecommons.org/licenses/by/4.0/), which permits unrestricted use, distribution, and reproduction in any medium,

A helpful spreadsheet detailing necessary calculations is included here and utilized multiple times throughout the protocol:

Example PPL-BPCA Calculations.xlsx

Expected Protocol Timeline: From start to finish, completion of this protocol for dissolved black carbon (DBC) analysis will take approximately one week. However, there are multiple points in the protocol where the preparative procedures can be paused and resumed at a later time. Before starting each step in the procedure, read ahead to make sure you have allocated sufficient time to complete each step before reaching a designated pause point. To achieve maximum sample preparation efficiency and data consistency, we recommend that samples are prepared in relevant batches, rather than one at a time. For example, solid phase extracted dissolved organic carbon (DOC) can be archived in the freezer until an entire sample set is collected, then the samples can be analyzed for DBC in one batch.

Chemical considerations: Analytical grade chemicals and solvents should be used at all times.

Cleaning glassware*:

1. Triple rinse with ultrapure or deionized water
2. Soak in 0.01M hydrochloric acid bath (ultrapure water acidified to pH 2) for 6+ hours
3. Triple rinse with ultrapure water
4. Place in a drying oven set to ~60°C until glassware is completely dry
5. Remove glassware from oven and cover any openings with aluminum foil
6. Combust foiled glassware in a muffle oven at 500°C for 5 hours

***Exceptions to this procedure include:**

- Glass ampules and HPLC vials, which are simply wrapped in foil and combusted (steps 5 and 6 only)
- HPLC vial inserts, which are used directly from the package
- Volumetric glassware, such as volumetric pipettes or flasks, should be cleaned following the "Cleaning plasticware" procedure below

Cleaning plasticware:

1. Triple rinse with ultrapure or deionized water
2. Soak in 0.01M hydrochloric acid bath (ultrapure water acidified to pH 2) for 6+ hours
3. Triple rinse with ultrapure water
4. Place in a drying oven set to ~60°C until plasticware is completely dry

Necessary materials are listed below, please see attached spreadsheet for more details.

DBC Quantification Materials List.xlsx

Instrumentation/Large Equipment:

- muffle oven: capable of heating glassware to 500ºC for 5 hours
- drying oven: oven that can maintain 60ºC (optional but beneficial for evaporating methanol)
- torch for flame sealing: capable of melting borosilicate glass

Citation: Riley Barton, Sasha Wagner Measuring dissolved black carbon in water via aqueous, inorganic, high performance liquid chromatography of benzenepolycarboxylic acid (BPCA) molecular markers https://dx.doi.org/10.17504/protocols.io.5qpvoy2b9g4o/v2

This is an open access protocol distributed under the terms of the **Creative Commons Attribution License** (https://creativecommons.org/licenses/by/4.0/), which permits unrestricted use, distribution, and reproduction in any medium,

- oxidation oven: programmable oven that can be set to 160ºC and hold steady for 6 hours
- HPLC-DAD: must be able to run a 2-solution buffer gradient and detect absorbance at 240nm

Chemicals:
6N Hydrochloric acid (Fischer SA56-4), Methanol (Fischer A4544), Acetone (Fischer A9294), 15.8N nitric acid (Fischer A200-212), 85% o-phosphoric acid (Fischer A260-500), sodium phosphate monohydrate (Fischer S369-500), sodium phosphate heptahydrate (Fischer S373-500), hemimellitic acid (TCI America H15925G), 1,3,5-benzenetricarboxylic acid (Thermo Scientific 105350500), 1,2,4-benzenetricarboxylic acid (Thermo Scientific 349030050), 1,2,4,5-benzenetetracarboxylic acid (Thermo Scientific 105311000), benzenepentacarboxylic acid (TCI America B095225G), benzenehexacarboxylic acid (TCI America B024625G)

Gases:
High purity nitrogen (Airgas NI HP300) ***or*** Argon (Airgas AR UHP300) ***and/or*** zero air (Airgas AI Z300)*
*zero air cannot be exclusively used, you must have either high purity nitrogen and/or argon for this protocol

HPLC columns & SPE cartridges:
phenyl-hexyl column 4.6 x 150 mm, 2.7 µm (InfinityLab Poroshell 120 693975-912), phenyl-hexyl guard column 4.6 x 5 mm, 2.7 µm (InfinityLab Poroshell 120 820750-914), Bond Elut PPL cartridge 1g, 6mL (Aligent 12255002)

Drying Apparatus Materials:
- Connectors/Adapters: one-way stopcock with male slip luer, adapter cap for 1, 3 and 6mL Bond Elut cartridges, five port manifold with female luer locks, male luer to hosebarb adapter (3/16"), male luer to male luer connector, female luer to hose barb adapter (3/32"), male luer lock to female luer,
- Tubing: 3/16in Pump Tubing (Masterflex L/S 25), Pump Tubing (Masterflex L/S 16), nylon tubing 4mm OD, 2.5mm ID (Shimadzu 016-43284-01)
- Other: sand, porcelain evaporating dishes, 5-gang valve aquarium pump accessory, hypodermic needles

Miscellaneous Materials:
- Nalgene 890 tubing (1/16X1/8X1/32), male luer lock to hosebarb adapter (1/16"), pre-scored ampules 2mL (Wheaton 176776), serum vial racks (23.8 x 11.1 x 8.9 cm), SPE tube caps, female luer caps, 12-mL glass vials, mechanical pipettes (20-200uL and 100-1000uL), glass Pasteur pipettes, 2-mL vial rack, long steel forceps, 9mm wide opening glass thread vials (2-mL), 9mm autosampler inserts (375 uL), 9 mm autosampler vial screw thread caps

Examine the SDS sheets for all chemicals before using. This protocol includes handling concentrated acids, organic solvent, gas tanks, and open flames. Please use appropriate personal protective equipment at all times.

Prior to sample preparation and DBC analysis, all water samples should be:

Citation: Riley Barton, Sasha Wagner Measuring dissolved black carbon in water via aqueous, inorganic, high performance liquid chromatography of benzenepolycarboxylic acid (BPCA) molecular markers https://dx.doi.org/10.17504/protocols.io.5qpvoy2b9g4o/v2

This is an open access protocol distributed under the terms of the **Creative Commons Attribution License** (https://creativecommons.org/licenses/by/4.0/), which permits unrestricted use, distribution, and reproduction in any medium,

- Collected and filtered using supplies cleaned according to the above protocol. During sampling, care should be taken to avoid sample contamination (e.g. rinsing sample bottle with sample before collection, collecting water upstream from your position, avoiding the introducing sunscreen/oils from hands to water or inner sample bottle, etc). Samples are typically filtered through pre-cleaned filters with pore sizes ranging from 0.1 to 0.7 µm. Store filtered (un-acidified) water samples in the dark at 4°C until further analysis or up to 48 hours.
- Analyzed to obtain DOC concentrations. These DOC concentration data are essential for determining volumes for accurate sample preparation and robust DBC quantification. In addition, DOC concentrations also allow for proper contextualization of DBC data obtained using this protocol.

All glassware and plasticware should be cleaned according to procedures described herein.

Solid Phase Extraction: Prepare and condition cartridges 1h

1 The solid phase extraction (SPE) cartridges used in this protocol (Agilent Bond Elut PPL cartridge, 1g, 6 mL) is packed with a styrene-divinylbenzene polymer resin that recovers high proportions of dissolved organic matter (DOM) on a per-carbon basis. The resin must first be conditioned to enable DOM extraction from filtered water.

To set up for conditioning of SPE cartridges, construct an apparatus that securely holds the cartridges upright above a solvent-compatible container.

Citation: Riley Barton, Sasha Wagner Measuring dissolved black carbon in water via aqueous, inorganic, high performance liquid chromatography of benzenepolycarboxylic acid (BPCA) molecular markers https://dx.doi.org/10.17504/protocols.io.5qpvoy2b9g4o/v2

This is an open access protocol distributed under the terms of the **Creative Commons Attribution License** (https://creativecommons.org/licenses/by/4.0/), which permits unrestricted use, distribution, and reproduction in any medium,

3

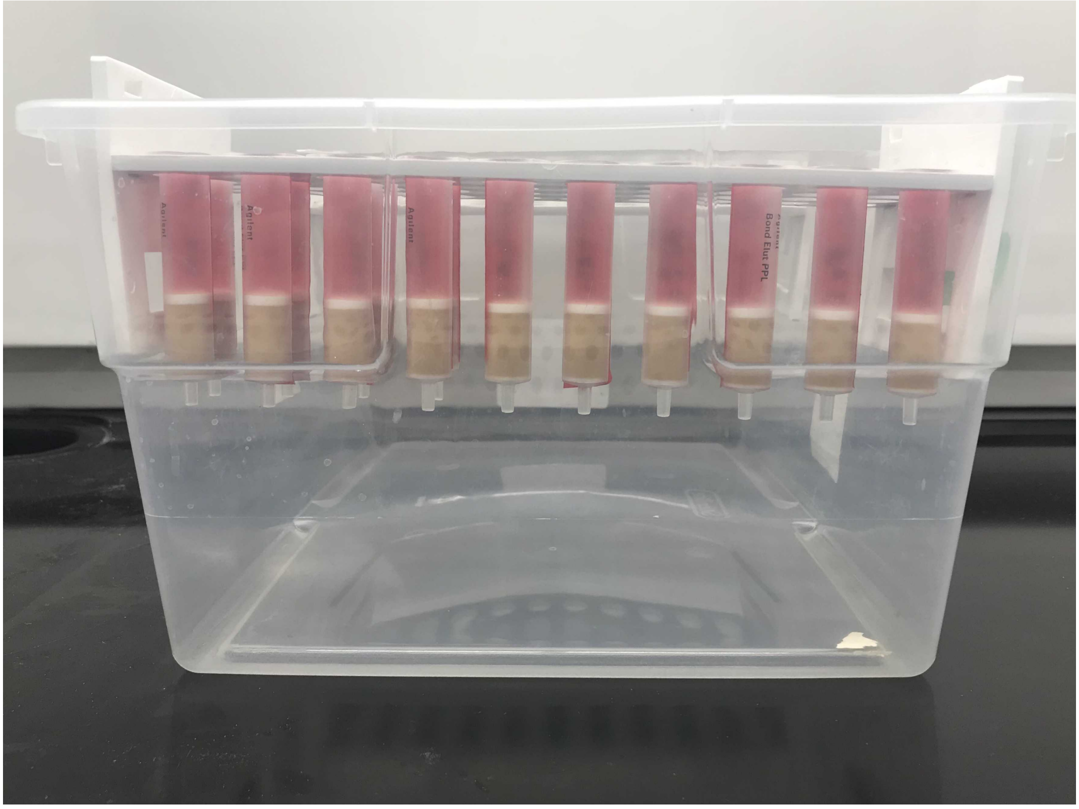

Image of our typical conditioning setup, which uses a test tube rack wedged into a plastic tub to hold and suspend SPE cartridges. Note that the backside of the pictured SPE cartridges have been labeled with red labeling tape, please see Step 5 for an image of un-labeled cartridges.

Dittmar T, Koch B, Hertkorn N, Kattner G. (2008). A simple and efficient method for the solid-phase extraction of dissolved organic matter (SPE-DOM) from seawater. Limnol. Oceanogr.: Meth. https://doi.org/10.4319/lom.2008.6.230

2 Condition SPE cartridges with analytical grade methanol (3 headspace volumes). 15m

Fill beaker with methanol, then transfer methanol from beaker to cartridge with a volumetric pipette. Allow methanol to flow through the cartridge until the meniscus is almost touching the white filter located above the resin. Repeat two more times and immediately proceed to next step.

3 Condition SPE cartridges with ultrapure water (3 headspace volumes). 15m

Citation: Riley Barton, Sasha Wagner Measuring dissolved black carbon in water via aqueous, inorganic, high performance liquid chromatography of benzenepolycarboxylic acid (BPCA) molecular markers https://dx.doi.org/10.17504/protocols.io.5qpvoy2b9g4o/v2

This is an open access protocol distributed under the terms of the **Creative Commons Attribution License** (https://creativecommons.org/licenses/by/4.0/), which permits unrestricted use, distribution, and reproduction in any medium,

Fill beaker with ultrapure water, then transfer ultrapure water from beaker to cartridge with a volumetric pipette. Allow ultrapure water to flow through the cartridge until the meniscus is almost touching the white filter located above the resin. Repeat two more times and immediately proceed to next step.

4 Condition SPE cartridges with pH 2 water (3 headspace volumes). 15m

For this step, the pH 2 water should be made fresh by adding 2 mL of 6 N hydrochloric acid (or 1 mL of 12 N hydrochloric acid) to 1 L of ultrapure water. To avoid contamination by organic material, it is important to use pre-cleaned glassware and to make up the pH 2 water solution just before use.

Transfer pH 2 water to cartridge with a volumetric pipette. Allow pH 2 water to flow through the cartridge until the meniscus is almost touching the white filter located above the resin. Repeat two more times and immediately proceed to next step.

5 Fill headspace of SPE cartridges with pH 2 water and immediately cap top and bottom.

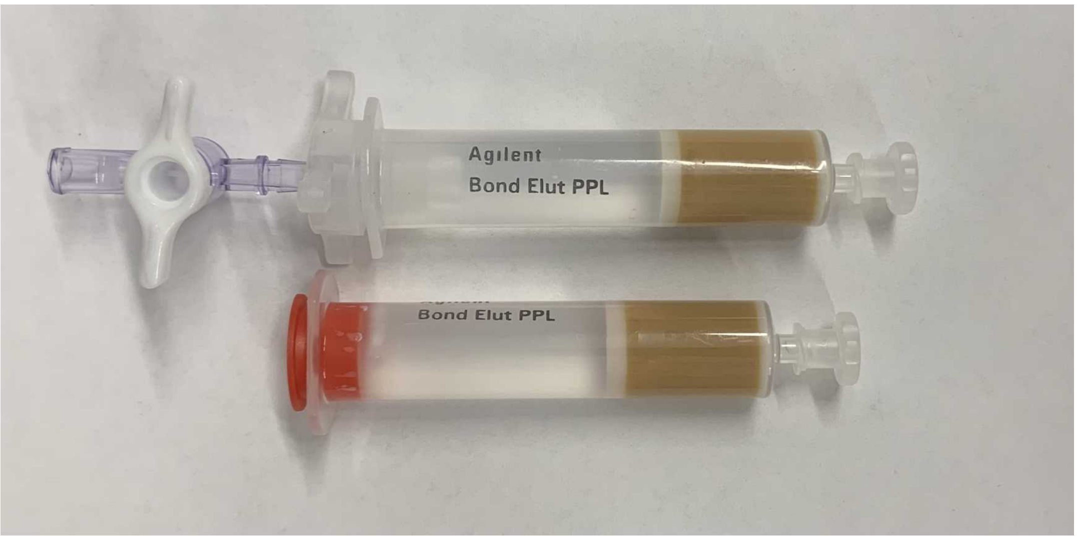

Two capped, conditioned SPE cartridges (1g, 6-mL). The top cartridge is capped using a female luer cap (Cole-Parmer EW-45501-28), an adapter cap (Agilent 12131001), and luer lock stopcock (Cole-Parmer EW-30600-06). The bottom cartridge is capped using a female luer cap (Cole-Parmer EW-45501-28) and a SPE tube cap (Millipore 52173-U).

Ensure there is little to no headspace in the cartridge before capping. It is important that the headspace is nearly filled with pH 2 water to ensure the cartridge remains conditioned and will not dry out before DOM extraction.

Citation: Riley Barton, Sasha Wagner Measuring dissolved black carbon in water via aqueous, inorganic, high performance liquid chromatography of benzenepolycarboxylic acid (BPCA) molecular markers https://dx.doi.org/10.17504/protocols.io.5qpvoy2b9g4o/v2

This is an open access protocol distributed under the terms of the **Creative Commons Attribution License** (https://creativecommons.org/licenses/by/4.0/), which permits unrestricted use, distribution, and reproduction in any medium,

6

Store SPE cartridges in the refrigerator at 4 °C until DOM extraction.

Do not store cartridges in the freezer as water expansion will cause the caps to come off and resin to dry out.

Conditioned SPE cartridges may be stored like this in the refrigerator for several weeks to months.

Solid Phase Extraction: Extraction of DOM 1d

7

At this point, the water sample should have been filtered and analyzed for dissolved organic carbon (DOC) concentration. In order to determine the volume of sample necessary to extract, please see the Estimating Sample Volume tab in the Example PPL-BPCA Calculations spreadsheet. In general, approximately 0.5-mg of extracted DOC is used to quantify DBC in the original sample. As extraction efficiencies and amount of DBC present are sample-dependent, we suggest passing more than the minimum volume through the cartridge and instead aim for a volume that will oxidize to ~20uM of benzenehexacarboxylic acid (B6CA). Please see linked spreadsheet and the rest of the protocol for further discussion and elaboration.

Example PPL-BPCA Calculations.xlsx

Once the necessary volume is determined, acidify the filtered water sample to pH 2 using hydrochloric acid.

Adding 2 mL of 6 N hydrochloric acid (or 1 mL of 12 N hydrochloric acid) to 1 L of filtered sample water usually brings the pH value close to 2. However, depending on sample composition and buffering capacities, additional hydrochloric acid may be needed to sufficiently reduce the pH.

After each hydrochloric acid addition, cap and vigorously mix the sample, then test the pH by dropping the solution onto a pH strip or by using a pH meter.

The acidification of the sample protonates molecules contained within DOM so they effectively sorb to the conditioned SPE resin. **The acidification step is critical for DOM**

Citation: Riley Barton, Sasha Wagner Measuring dissolved black carbon in water via aqueous, inorganic, high performance liquid chromatography of benzenepolycarboxylic acid (BPCA) molecular markers https://dx.doi.org/10.17504/protocols.io.5qpvoy2b9g4o/v2

This is an open access protocol distributed under the terms of the **Creative Commons Attribution License** (https://creativecommons.org/licenses/by/4.0/), which permits unrestricted use, distribution, and reproduction in any medium,

recovery.

8

Record the mass of sample bottle containing the acidified sample.

Recording this mass is necessary for determining the actual volume of sample extracted.

If you do not have access to an appropriate balance, then the volume of sample extracted can also be determined by measuring the volume of SPE effluent using a graduated cylinder.

9

Set up SPE apparatus and begin flow of acidified sample through SPE cartridge.

Citation: Riley Barton, Sasha Wagner Measuring dissolved black carbon in water via aqueous, inorganic, high performance liquid chromatography of benzenepolycarboxylic acid (BPCA) molecular markers https://dx.doi.org/10.17504/protocols.io.5qpvoy2b9g4o/v2

This is an open access protocol distributed under the terms of the **Creative Commons Attribution License** (https://creativecommons.org/licenses/by/4.0/), which permits unrestricted use, distribution, and reproduction in any medium,

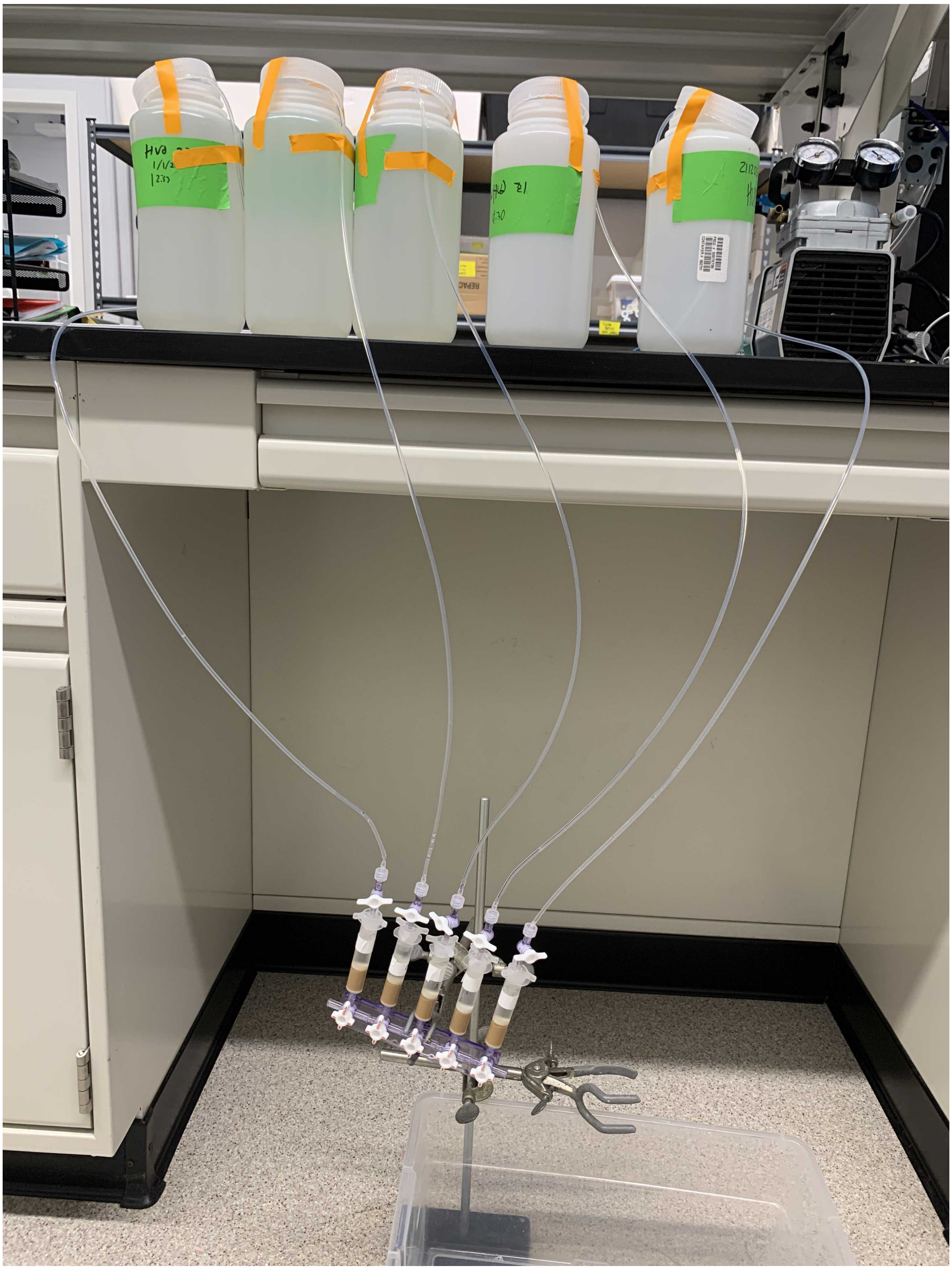

Image of SPE apparatus for 5 samples.

9.1 Insert open end of Teflon tube into sample bottle until it touches the bottom (stopcock should be closed at this point). Tape the Teflon tube to the outside of the bottle to keep it in place during the extraction process.

The Teflon tubing used in this step is cut to ~0.5-1 m in length and a luer lock

Citation: Riley Barton, Sasha Wagner Measuring dissolved black carbon in water via aqueous, inorganic, high performance liquid chromatography of benzenepolycarboxylic acid (BPCA) molecular markers https://dx.doi.org/10.17504/protocols.io.5qpvoy2b9g4o/v2

This is an open access protocol distributed under the terms of the **Creative Commons Attribution License** (https://creativecommons.org/licenses/by/4.0/), which permits unrestricted use, distribution, and reproduction in any medium,

stopcock is fixed to one end using a male luer lock to hosebarb adapter piece. The length of the Teflon tube can be modified depending upon the lab space. If multiple Teflon tubes are prepared for extractions, then multiple samples can be extracted concurrently.

- Teflon tubing: (1/16X1/8X1/32) Nalgene 890 tubing (Thermo Scientific 8050-0125)
- luer lock stopcock (Cole-Parmer EW-30600-06)
- male luer lock to hosebarb adapter 1/16" (Masterflex HV-45518-00)

9.2 Cover sample bottle opening (e.g. with aluminum foil). Place sample bottle on a shelf, or other high and stable area, that will be located above the SPE cartridge.

9.3 Open stopcock to allow sample water to flow through it by gravity. Allow 5 to 10 mL of sample to flow out to waste, then close the stopcock.

This step begins flow, but also rinses the interior of the Teflon tube with sample before connection to the conditioned SPE cartridge.

If sample water does not begin flowing from gravity alone, you may use a syringe to gently apply vacuum to the stopcock end of the Teflon tube to initiate flow.

9.4 Connect Teflon tubing from sample bottle to SPE cartridge via the following steps:

1. Insert stopcock into a plastic adapter cap
2. Label body of SPE cartridge with sample identifier or name
3. Remove SPE cartridge caps and place into manifold (all stopcocks should be closed to prevent flow)
4. Open stopcock and let sample drip into SPE cartridge until headspace is nearly full
5. Close stopcock and snap adapter cap to the top of the SPE cartridge

Repeat steps above for each sample until the manifold is full.

Citation: Riley Barton, Sasha Wagner Measuring dissolved black carbon in water via aqueous, inorganic, high performance liquid chromatography of benzenepolycarboxylic acid (BPCA) molecular markers https://dx.doi.org/10.17504/protocols.io.5qpvoy2b9g4o/v2

This is an open access protocol distributed under the terms of the **Creative Commons Attribution License** (https://creativecommons.org/licenses/by/4.0/), which permits unrestricted use, distribution, and reproduction in any medium,

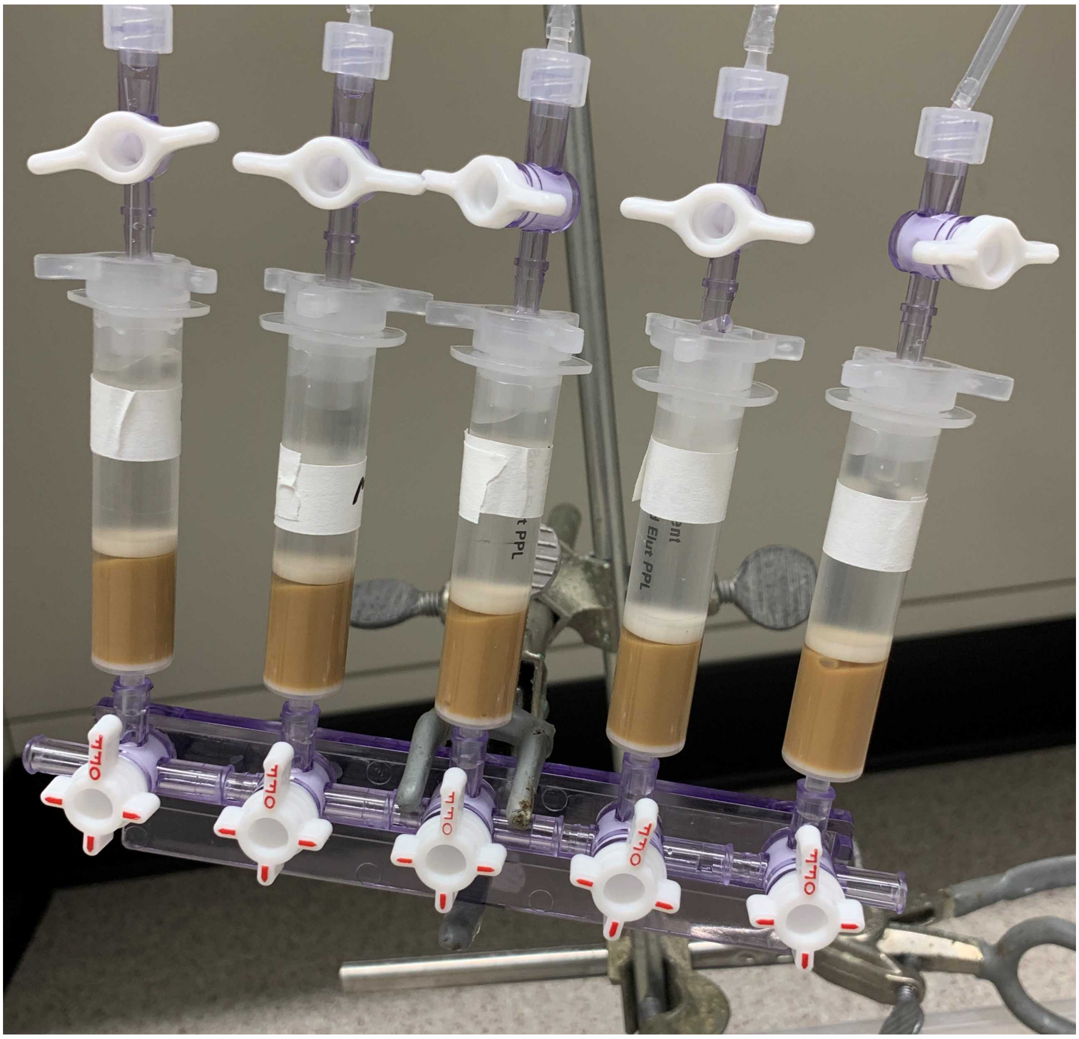

Image of full manifold.

Conditioned SPE cartridges should be filled with ultrapure water (pH = 2) from conditioning steps. Before placing them in the manifold, ensure that they are still filled and that the resin has not dried out. There is no need to drain conditioning fluid before extracting sample.

10 Open each stopcock sequentially, checking to make sure sample is flowing to and effluent is flowing from each cartridge and that flow is exiting the manifold.

You can also double check for sample acidification by letting the effluent drip onto pH paper.

Citation: Riley Barton, Sasha Wagner Measuring dissolved black carbon in water via aqueous, inorganic, high performance liquid chromatography of benzenepolycarboxylic acid (BPCA) molecular markers https://dx.doi.org/10.17504/protocols.io.5qpvoy2b9g4o/v2

This is an open access protocol distributed under the terms of the **Creative Commons Attribution License** (https://creativecommons.org/licenses/by/4.0/), which permits unrestricted use, distribution, and reproduction in any medium,

11 Place an appropriately sized container beneath the manifold to capture SPE effluent and wait until extraction is complete (i.e. the entire sample volume has passed through the SPE cartridge by gravity).

Depending upon sample volume, sample composition and/or flow rate, this extraction step can take anywhere from a few hours to a full day to complete. It is typical to set up extractions in the afternoon, then leave the extraction to continue overnight and complete the following steps the following day.

Possible issues during extraction:

- **Small sample bottles** - It may be necessary to secure the sample bottles on the shelf, as they may be displaced by the weight of the tubes after sample is drained from the bottle. This is typically a problem for smaller bottle sizes (e.g., less than 1 L) and should be considered during extraction setup.

- **Bubbles in headspace** - Formation of bubbles can introduce air to the resin and slow the flow rate of sample. If small bubbles form in the SPE cartridge headspace, gently tap the side of the cartridge with a pen or similar object. If you observe the formation of large bubbles or major gaps of air in the headspace, remove adapter cap connection and allow sample to flow and refill headspace of cartridge, then reattach adapter.

- **Flocculation of DOM** - If there are no bubbles present and flow through SPE cartridge is slow, check to see if the white filter (above resin) has turned brown and/or accumulated sample particulates. In highly concentrated samples (e.g., samples collected from a wetland or during peak river discharge), DOM may flocculate out of solution when acidified to pH 2 and clog the SPE cartridge. If flow is slow, but still dripping, leave samples to extract overnight or for 24 hours. If flow has stopped completely or is prohibitively slow, then you may use another SPE cartridge to extract DOM from the remaining volume. Depending upon sample DOC concentration and expected SPE recovery, the volume of sample extracted prior to clogging may be sufficient for further analyses.

12 Once extractions are complete, close stopcocks and remove tubing and adapter caps from the sample bottles and SPE cartridges.

13 ⚠

Record the mass of the empty sample bottle.

Citation: Riley Barton, Sasha Wagner Measuring dissolved black carbon in water via aqueous, inorganic, high performance liquid chromatography of benzenepolycarboxylic acid (BPCA) molecular markers https://dx.doi.org/10.17504/protocols.io.5qpvoy2b9g4o/v2

This is an open access protocol distributed under the terms of the **Creative Commons Attribution License** (https://creativecommons.org/licenses/by/4.0/), which permits unrestricted use, distribution, and reproduction in any medium,

Recording this mass is necessary for determining the actual volume of sample extracted.

Note that there will likely be some sample remaining in the sample bottle.

If you do not have access to an appropriate balance, then the volume of sample extracted can also be determined by measuring the volume of SPE effluent using a graduated cylinder.

14 Rinse SPE cartridges with pH 2 water (3 headspace volumes).

At this point, the SPE cartridges have sample DOM sorbed to the resin. This step desalts the sample by rinsing off potentially interfering inorganic material that may be stuck to the resin.

For this step, the pH 2 water should be made fresh by adding 2 mL of 6 N hydrochloric acid (or 1 mL of 12 N hydrochloric acid) to 1 L of ultrapure water. To avoid contamination by organic material, it is important to use pre-cleaned glassware and to make up the pH 2 water solution just before use.

Transfer pH 2 water to cartridge with a volumetric pipette. Allow pH 2 water to flow through the cartridge until the meniscus is almost touching the white filter located above the resin. Repeat two more times.

15

At this point, you may continue directly on to the next step or recap DOM-containing SPE cartridges and wrap in aluminum foil and freeze/refrigerate for up to three months before continuing on to the next step.

Solid Phase Extraction: Drying SPE cartridges 1h

16 Set up the drying apparatus and attach DOM-containing SPE cartridges via the following steps:

1. Suspend and secure manifolds using a clamp and ring stand
2. Use tubing and connectors to attach manifolds to gas tank*
3. Attach SPE cartridge to manifold using an adapter cap connector
4. Lay paper towels or other absorbent material beneath the manifold and cartridges

*Any of the following gases may be used to dry SPE cartridges: high purity nitrogen, argon, or zero air.

Citation: Riley Barton, Sasha Wagner Measuring dissolved black carbon in water via aqueous, inorganic, high performance liquid chromatography of benzenepolycarboxylic acid (BPCA) molecular markers https://dx.doi.org/10.17504/protocols.io.5qpvoy2b9g4o/v2

This is an open access protocol distributed under the terms of the **Creative Commons Attribution License** (https://creativecommons.org/licenses/by/4.0/), which permits unrestricted use, distribution, and reproduction in any medium,

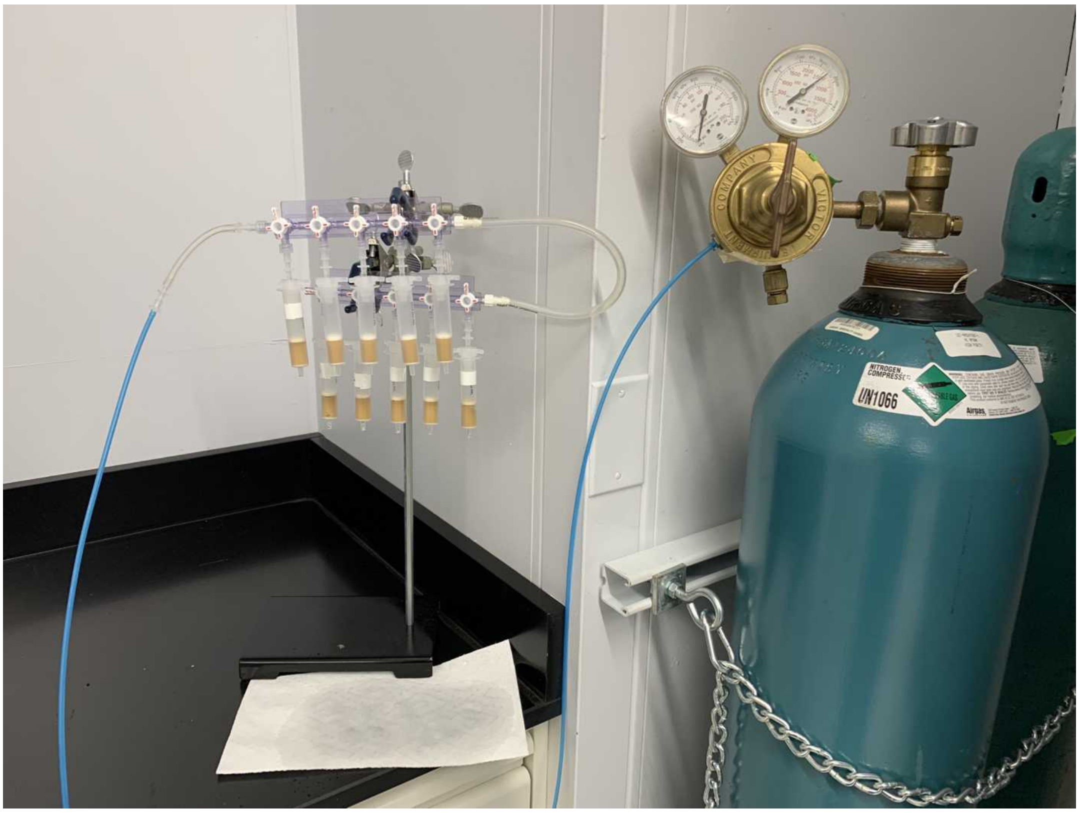

Image of a possible drying apparatus setup.

Make sure manifold valves are set so that gas will flow freely through every SPE cartridge and no gas flow escapes through other manifold openings.

We use a combination of the following connectors, tubing, and adapters to construct the drying apparatus shown here:

- tubing: nylon tubing 4mm OD, 2.5mm ID (Shimadzu 016-43284-01), precision pump tubing L/S 25, 4.8mm (Masterflex ZY-96410-25), precision pump tubing L/S 16, 3.1mm (Masterflex ZY-96410-16)
- connectors: Male luer to male luer (Masterflex HV-30800-14), male luer to hosebarb adapter 3/16" (Masterflex ZY-45505-08), male luer lock to female luer (Masterflex ZY-45502-80), female luer to hosebarb 3/32" (Masterflex ZY-45501-02)
- adapters: Five port manifold with female luer locks (Masterflex HV-30600-43), adapter cap for 1,3,6mL cartridges (Agilent 12131001)

However, other materials and configurations may be used to construct a drying apparatus that works best for your lab setup.

17 Turn on gas flow.

Citation: Riley Barton, Sasha Wagner Measuring dissolved black carbon in water via aqueous, inorganic, high performance liquid chromatography of benzenepolycarboxylic acid (BPCA) molecular markers https://dx.doi.org/10.17504/protocols.io.5qpvoy2b9g4o/v2

This is an open access protocol distributed under the terms of the **Creative Commons Attribution License** (https://creativecommons.org/licenses/by/4.0/), which permits unrestricted use, distribution, and reproduction in any medium,

Keep gas flow relatively high at first, until water stops spitting rapidly from the SPE cartridges. Then reduce flow to a point where you can just feel it on the back of your hand when held near the outflow of the SPE cartridge. Here, you want to find a balance that minimizes both drying time and gas waste. This will take some trial and error to determine the appropriate flow rate for your specific drying setup.

18 Allow gas to flow until SPE cartridge resin is completely dry. 1h

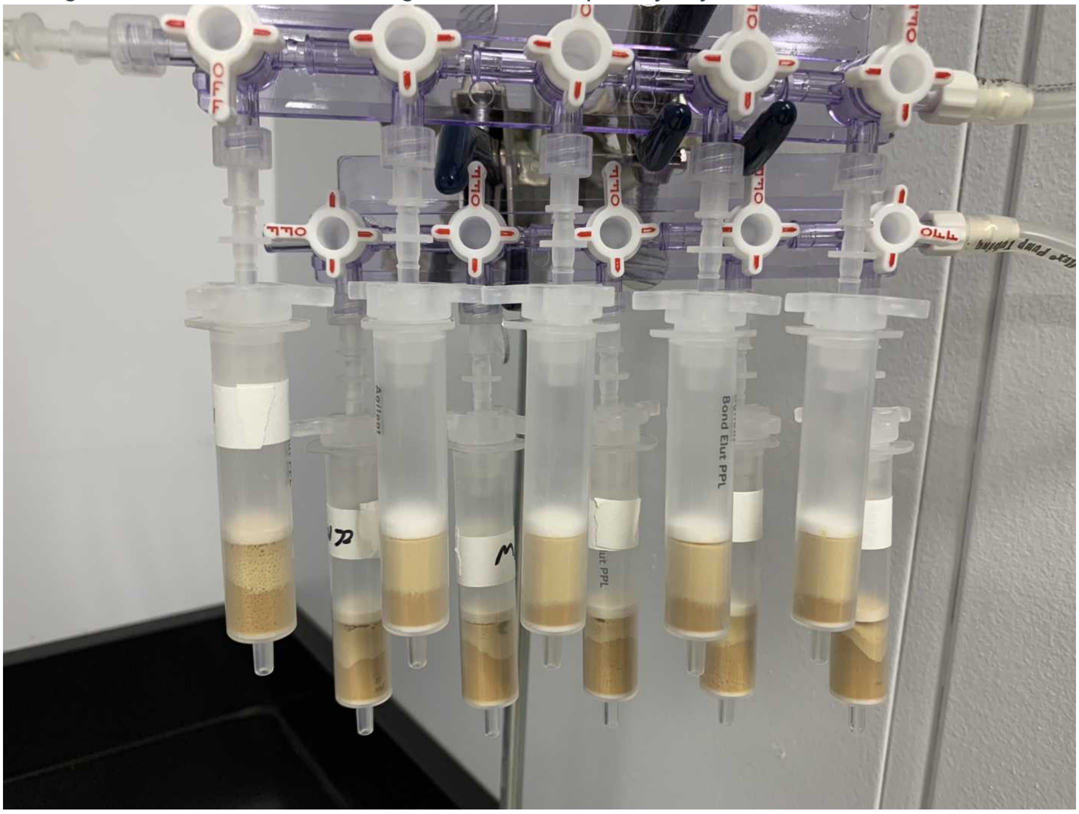

Image of SPE cartridges with partially-dry resin.

Dry resin has a lighter color and feels room temperature to the touch when gas is flowing through columns. If you are unsure whether the resin is dry, unhook and tap the side of the cartridge – The resin is dry when it moves freely, like dry sand, in the cartridge.

Since the resin swells slightly when wet, you may observe extra space in the resin area (between the two white filters) after extraction and drying - This is normal and will not affect the next extraction steps.

Citation: Riley Barton, Sasha Wagner Measuring dissolved black carbon in water via aqueous, inorganic, high performance liquid chromatography of benzenepolycarboxylic acid (BPCA) molecular markers https://dx.doi.org/10.17504/protocols.io.5qpvoy2b9g4o/v2

This is an open access protocol distributed under the terms of the **Creative Commons Attribution License** (https://creativecommons.org/licenses/by/4.0/), which permits unrestricted use, distribution, and reproduction in any medium,

19 Turn off gas at the tank and remove dry SPE cartridges from manifolds. Repeat drying process as necessary to dry the rest of the SPE cartridges in the sample set.

20

Remember to turn off gas when you are done. If you do not have time to move on to the next step, cap the SPE cartridges and wrap in aluminum foil until you are ready to elute the DOM. Cartridges should be stored at 4°C.

Solid Phase Extraction: Elution of SPE-DOC with methanol 2h

21 Label glass vials with sample identifier or name.

> Here, we typically use 12 mL glass vials with Teflon-lined caps. The 12 mL volume minimizes vial headspace and takes up less freezer space if a large number of samples are being collected and archived.
>
> We suggest typing or writing sample identifiers on paper and then using clear tape to fix labels to vials. Spilled methanol or solvent fumes will dissolve permanent marker and distort certain adhesive labels.

22 ⚠

Record the mass of the empty glass vial (with cap on).

> Recording this mass is necessary for determining the actual volume of methanol used to elute DOM from the SPE cartridge.

23 Using a test tube rack, or similar apparatus, suspend the SPE cartridges in preparation for DOM elution. Remove caps from vials and place them underneath their corresponding SPE cartridges.

> This setup is meant to facilitate elution of DOM in methanol from the SPE cartridge directly into the glass vial to ensure no DOM is lost during transfer. The outlet tips of the SPE cartridges should hover just above the opening of the glass vials to ensure that no drips of sample eluate miss the vial. **It is essential that you collect the first few drops** when

Citation: Riley Barton, Sasha Wagner Measuring dissolved black carbon in water via aqueous, inorganic, high performance liquid chromatography of benzenepolycarboxylic acid (BPCA) molecular markers https://dx.doi.org/10.17504/protocols.io.5qpvoy2b9g4o/v2

This is an open access protocol distributed under the terms of the **Creative Commons Attribution License** (https://creativecommons.org/licenses/by/4.0/), which permits unrestricted use, distribution, and reproduction in any medium,

eluting DOM with methanol, as these first drops are the most concentrated with DOM.

Keep vial caps organized so the correct cap can be placed on the corresponding vial after elution. This is necessary to ensure accurate quantification of methanol volumes since caps vary slightly in their measured mass.

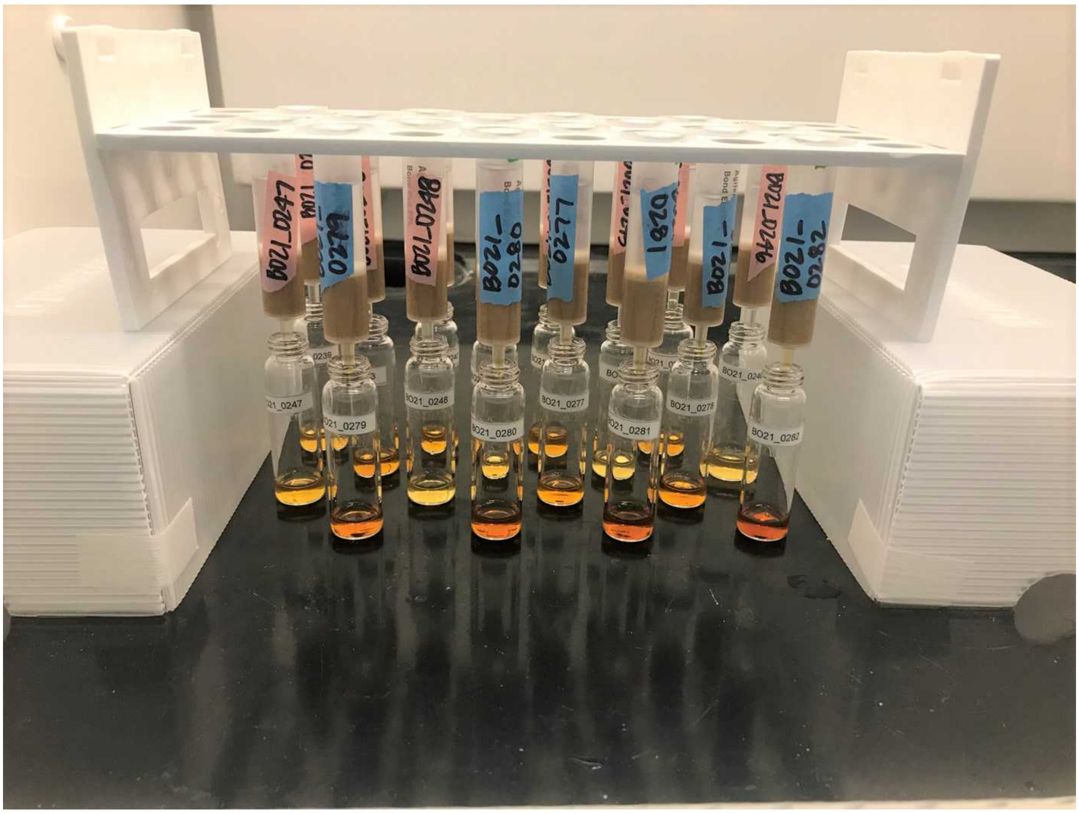

Image of elution apparatus with samples mid-elution.

24 In this step, DOM is eluted from the SPE resin with methanol and collected directly in a glass vial. 1h
It is important to use analytical grade methanol so as not to contaminate the DOM sample with extraneous material.

Elute DOM from the SPE resin by adding 3 x 3 mL aliquots of methanol to the cartridge.

Fill beaker with methanol, then transfer ~3 mL of methanol from beaker to cartridge with a volumetric pipette. Allow methanol to flow through the cartridge until the meniscus is almost touching the white filter located above the resin. Repeat two more times, letting the final 3 mL aliquot of methanol drain completely from the cartridge into the vial.

The total volume of methanol used to elute DOM from the SPE cartridges described here (Agilent Bond Elut PPL cartridge, 1g, 6 mL) is ~9 mL, but this volume may be adjusted to accommodate different resin masses and/or cartridge headspaces.

Citation: Riley Barton, Sasha Wagner Measuring dissolved black carbon in water via aqueous, inorganic, high performance liquid chromatography of benzenepolycarboxylic acid (BPCA) molecular markers https://dx.doi.org/10.17504/protocols.io.5qpvoy2b9g4o/v2

This is an open access protocol distributed under the terms of the **Creative Commons Attribution License** (https://creativecommons.org/licenses/by/4.0/), which permits unrestricted use, distribution, and reproduction in any medium,

25 Once flow from the SPE cartridges has stopped, close each vial with their corresponding cap.

26

Record the mass of the eluate-filled glass vial (with cap on).

Recording this mass is necessary for determining the actual volume of methanol used to elute DOM from the SPE cartridge.

27

At this point, you may continue directly to the next step or store eluate in the freezer until you are ready to continue. This methanol eluate can be stored for years in the freezer as an archive of SPE-DOC for future BPCA analyses.

Note that each SPE cartridge may be used up to three times before disposal. After DOM elution, SPE cartridges can be rinsed with analytical grade acetone (3 headspace volumes) and set aside to air dry. Once dry, the SPE cartridges can be reconditioned (see steps 1 though 6) and reused for DOM extraction. We recommend that each SPE cartridge is reused with a similar sample type so as to avoid potential cross contamination. For example, do not use an SPE cartridge to first extract a high DOC swamp water sample, then extract a low DOC open ocean sample. It is best when SPE cartridges are reused within the same sample set or for samples collected at the same field site.

Sample Oxidation: Transfer of SPE-DOC eluate to ampules 1h

28 Using the spreadsheet linked below (PPL tab), calculate the volume of DOM eluate that should be dried down for DBC analysis.

Example PPL-BPCA Calculations.xlsx

For robust DBC analysis, we recommend oxidizing ~0.5 mg of solid phase extracted DOC (SPE-DOC). By entering the following values into the spreadsheet, you will calculate the volume of sample passed through the SPE cartridge, the volume of methanol

Citation: Riley Barton, Sasha Wagner Measuring dissolved black carbon in water via aqueous, inorganic, high performance liquid chromatography of benzenepolycarboxylic acid (BPCA) molecular markers https://dx.doi.org/10.17504/protocols.io.5qpvoy2b9g4o/v2

This is an open access protocol distributed under the terms of the **Creative Commons Attribution License** (https://creativecommons.org/licenses/by/4.0/), which permits unrestricted use, distribution, and reproduction in any medium,

eluate recovered from the SPE cartridge, and the volume of DOM-containing methanol eluate that needs to be dried down for sample oxidation and analysis:

- Sample DOC concentration (µM)
- Mass of full sample bottle (g)
- Mass of empty sample bottle (g)
- Mass of empty glass vial (g)
- Mass of full glass vial (g)

Example calculations are included in the spreadsheet.

Assumptions made - The calculations used here assume 60% recovery of bulk DOC from river and other freshwater samples and 45% recovery from coastal marine and open ocean samples during solid phase extraction (Dittmar et al., 2008). The percent recovery of SPE-DOC should be adjusted according to the type of water sample being extracted. If you are unsure of what the SPE-DOC recovery is for a specific sample type, you may want to measure it directly. Otherwise, we suggest 50% as a good starting point until you become more familiar with the sample composition.

What if I have less than 0.5 mg SPE-DOC? Smaller amounts of SPE-DOC may be used for DBC analysis, but this is highly dependent upon the amount and quality of DBC that is expected for a given sample based upon preliminary data or known biogeochemical factors. See Wagner et al. (2017) and the main manuscript text for further discussion on this. When working with a new or biogeochemically unknown sample set, we strongly recommend starting with 0.5 mg SPE-DOC until you become more familiar with the limitations of your analytical setup and sample DBC composition.

Dittmar T, Koch B, Hertkorn N, Kattner G. (2008). A simple and efficient method for the solid-phase extraction of dissolved organic matter (SPE-DOM) from seawater. Limnol. Oceanogr.: Meth. https://doi.org/10.4319/lom.2008.6.230

Wagner S, Brandes J, Goranov AI, Drake TW, Spencer RGM, Stubbins A. (2018). Online quantification and compound-specific stable isotopic analysis of black carbon in environmental matrices via liquid chromatography-isotope ratio mass spectrometry. Limnol. Oceanogr.: Meth. https://doi.org/10.1002/lom3.10219

Citation: Riley Barton, Sasha Wagner Measuring dissolved black carbon in water via aqueous, inorganic, high performance liquid chromatography of benzenepolycarboxylic acid (BPCA) molecular markers https://dx.doi.org/10.17504/protocols.io.5qpvoy2b9g4o/v2

This is an open access protocol distributed under the terms of the **Creative Commons Attribution License** (https://creativecommons.org/licenses/by/4.0/), which permits unrestricted use, distribution, and reproduction in any medium,

29 Label glass ampules

Glass ampules should be labeled (written on) directly with permanent marker. Paper or adhesive labels may burn or be removed in the oxidation oven.

30 1h

Using a mechanical pipette, fitted with a glass Pasteur pipette tip, quantitatively transfer the calculated volume of methanol eluate ("V MeOH to dry" in spreadsheet) to the corresponding ampule.

Triple-rinse the glass pipette tip with fresh methanol before use and between each sample to prevent cross contamination. Dispose of each rinsing to the appropriate solvent waste container.

Mechanical pipette setup - Here, we use a small piece of silicone tubing to fit the glass Pasteur pipette tip to the mechanical pipette. Using the glass pipette prevents possible contamination of organic material from plastic pipette tips.

Quantitative transfer technique - When transferring the methanol eluate, insert tip of Pasteur pipette into methanol eluate and slowly pump up and down 3 to 5 times until the headspace in the Pasteur pipette is equilibrated and bubbles are no longer emitted from the pipette tip. After equilibrating, draw in the target amount of methanol eluate and transfer to the ampule. When transferring, place the pipette tip all the way down the neck of the ampule and try to make sure the eluate is deposited directly to the bottom of the ampule. Gently pump the pipette 3 to 5 times to ensure the total volume of methanol eluate is expelled into the ampule.

What if the "V MeOH to dry" volume is greater than 2 mL? Each ampule can contain up to 2 mL of methanol eluate. If the volume of eluate that needs to be dried down exceeds 2 mL, then we suggest transferring and drying the eluate in aliquots of 2 mL or less (see next step). Do not overfill the ampules, as this will cause the SPE-DOC to dry on the neck of the ampule and may interfere with analysis later.

Sample Oxidation: Evaporation of methanol from SPE-DOC eluate 1d

31 1d

Citation: Riley Barton, Sasha Wagner Measuring dissolved black carbon in water via aqueous, inorganic, high performance liquid chromatography of benzenepolycarboxylic acid (BPCA) molecular markers https://dx.doi.org/10.17504/protocols.io.5qpvoy2b9g4o/v2

This is an open access protocol distributed under the terms of the **Creative Commons Attribution License** (https://creativecommons.org/licenses/by/4.0/), which permits unrestricted use, distribution, and reproduction in any medium,

In this step methanol is evaporated completely from the eluate to obtain the dry SPE-DOC residue for oxidation.

Place ampules in a properly vented drying oven set to 🌡 **60 °C** . Loosely place a Kimwipe over the ampule openings to prevent contamination from extraneous material during drying. Depending upon the eluate volume, methanol evaporation can take anywhere from 12 to 36 hours.

> If you are unsure whether methanol is completely evaporated, then place the ampules back in the oven and dry for a few more hours. **The reaction between concentrated nitric acid and excess methanol is explosive, therefore complete evaporation of methanol before moving on to the next step is essential.**

Sample Oxidation: Nitric acid addition and sealing ampules 15m

32 **Do not begin this step if you do not have the time to flame-seal ampules and perform sample oxidation immediately thereafter.**

Using a volumetric, mechanical pipette, add 0.5 mL of analytical grade nitric acid (15.8N) to each ampule.

> During nitric acid addition, visually examine the ampules to determine whether there is any SPE-DOC residue in the ampule neck. If there is any visible SPE-DOC in the neck (methanol eluate can creep up the sides of the ampule during drying and deposit SPE-DOC), rinse it down with nitric acid.

33 ⚠

Flame seal the glass ampules.

> **If you are new to sealing glass ampules with an open flame torch, practice first with empty ampules.** Do not attempt to seal ampules containing critical samples until you are comfortable and confident with the flame sealing technique. Inadequate sealing of samples will result in sample loss during oxidation. **Please see the online version of this protocol for a video of ampule-sealing technique.**

Citation: Riley Barton, Sasha Wagner Measuring dissolved black carbon in water via aqueous, inorganic, high performance liquid chromatography of benzenepolycarboxylic acid (BPCA) molecular markers https://dx.doi.org/10.17504/protocols.io.5qpvoy2b9g4o/v2

This is an open access protocol distributed under the terms of the **Creative Commons Attribution License** (https://creativecommons.org/licenses/by/4.0/), which permits unrestricted use, distribution, and reproduction in any medium,

33.1 The flame sealing of ampules requires a beaker of water, long metal forceps, safety glasses, and a blow torch that is sufficiently hot enough to melt borosilicate glass.

33.2 Turn on the torch. Hold ampule at a 45 degree angle. Use your hand to stabilize the base of the ampule and the forceps to stabilize the neck of the ampule.

33.3 Hold the upper neck area of the ampule in the flame and slowly rotate until the glass begins to soften. Once the glass is sufficiently malleable, gently rotate and pull apart the top and bottom portions of the ampule by pulling the forceps further away from the flame until the top portion is removed and the neck of the ampule is pinched closed. After removing the top part of the ampule, hold the sealed portion in the flame for an extra second or so to ensure a good seal.

Do not twist the glass too quickly, or before the glass is sufficiently malleable, as this may result in a poor seal. Rotate the bottom of the ampule with your hand and gently open and close the forceps to obtain the best seal.

33.4 Place the top piece of the ampule (pulled off using forceps) into the beaker of water to cool. Carefully place sealed ampule into a tray using the forceps.

Freshly sealed ampules are HOT - Move them using the forceps until they are cool to the touch.

34 Examine the ampule for a proper seal and repeat the previous step for each ampule in the sample set.

The sealed end of the ampule should resemble a candle flame. If you notice any holes in the seal, you may attempt to reseal using the same method described above. Note that it is more difficult to seal an ampule on the second try because the neck area is considerably shorter.

Citation: Riley Barton, Sasha Wagner Measuring dissolved black carbon in water via aqueous, inorganic, high performance liquid chromatography of benzenepolycarboxylic acid (BPCA) molecular markers https://dx.doi.org/10.17504/protocols.io.5qpvoy2b9g4o/v2

This is an open access protocol distributed under the terms of the **Creative Commons Attribution License** (https://creativecommons.org/licenses/by/4.0/), which permits unrestricted use, distribution, and reproduction in any medium,

35

Wrap ampules in aluminum foil pouches, usually 3 to 5 ampules per pouch.

While not completely necessary, wrapping ampules in foil helps organize large numbers of ampules during oxidation. In the event of an ampule leak or breakage, wrapping ampules can maintain the integrity and readability of ampule labels. If you are unsure whether a particular ampule has a strong seal, you may want to wrap that ampule individually. If analyzing replicates, you may choose to wrap replicate ampules together.

36

Place pouches together in a glass beaker or other glass container and place in a temperature controlled oven.

This step is optional, but it makes it easier to transfer ampules in and out of the oven safely and efficiently.

37

Oxidize samples at **160 °C** for 6 hours.

For further discussion on how these oxidation conditions were selected, see Ding et al. (2013).

If you are using a programmable oven, program the oven to ramp from 25°C to 160°C in 5°C per minute increments, then hold at 160°C for 6 hours before cooling to room temperature. In our lab, we use a decommissioned gas chromatograph oven to carry out sample oxidations.

If you are not using a programmable oven, set the oven to 160°C and wait for it to come to temperature, then add ampules and set a timer for 6 hours before removing them.

Citation: Riley Barton, Sasha Wagner Measuring dissolved black carbon in water via aqueous, inorganic, high performance liquid chromatography of benzenepolycarboxylic acid (BPCA) molecular markers https://dx.doi.org/10.17504/protocols.io.5qpvoy2b9g4o/v2

This is an open access protocol distributed under the terms of the **Creative Commons Attribution License** (https://creativecommons.org/licenses/by/4.0/), which permits unrestricted use, distribution, and reproduction in any medium,

Ding Y, Yamashita Y, Dodds WK, Jaffé R. (2013). Dissolved black carbon in grassland streams: is there an effect of recent fire history?. Chemosphere.
https://doi.org/10.1016/j.chemosphere.2012.10.098

38

Note that you must be present when the oxidation finishes to transfer ampules from the oven. This is especially important if you are using a non-programmable oven that does not automatically cool after the oxidation period.

After oxidation is complete, you may immediately move on to the next step or place fully cooled ampules into a freezer up to one week before continuing to the next step.

HPLC Analysis of BPCAs: Evaporation of nitric acid from ampules 2h

39 At this point, the sealed glass ampules contain sample benzenepolycarboxylic acids (BPCAs) and other SPE-DOC oxidation products dissolved in the concentrated nitric acid. In this step, the concentrated nitric acid is removed through evaporation under a stream of inert gas so the BPCAs can be prepared for separation and quantification by high performance liquid chromatography (HPLC).

Citation: Riley Barton, Sasha Wagner Measuring dissolved black carbon in water via aqueous, inorganic, high performance liquid chromatography of benzenepolycarboxylic acid (BPCA) molecular markers https://dx.doi.org/10.17504/protocols.io.5qpvoy2b9g4o/v2

This is an open access protocol distributed under the terms of the **Creative Commons Attribution License** (https://creativecommons.org/licenses/by/4.0/), which permits unrestricted use, distribution, and reproduction in any medium,

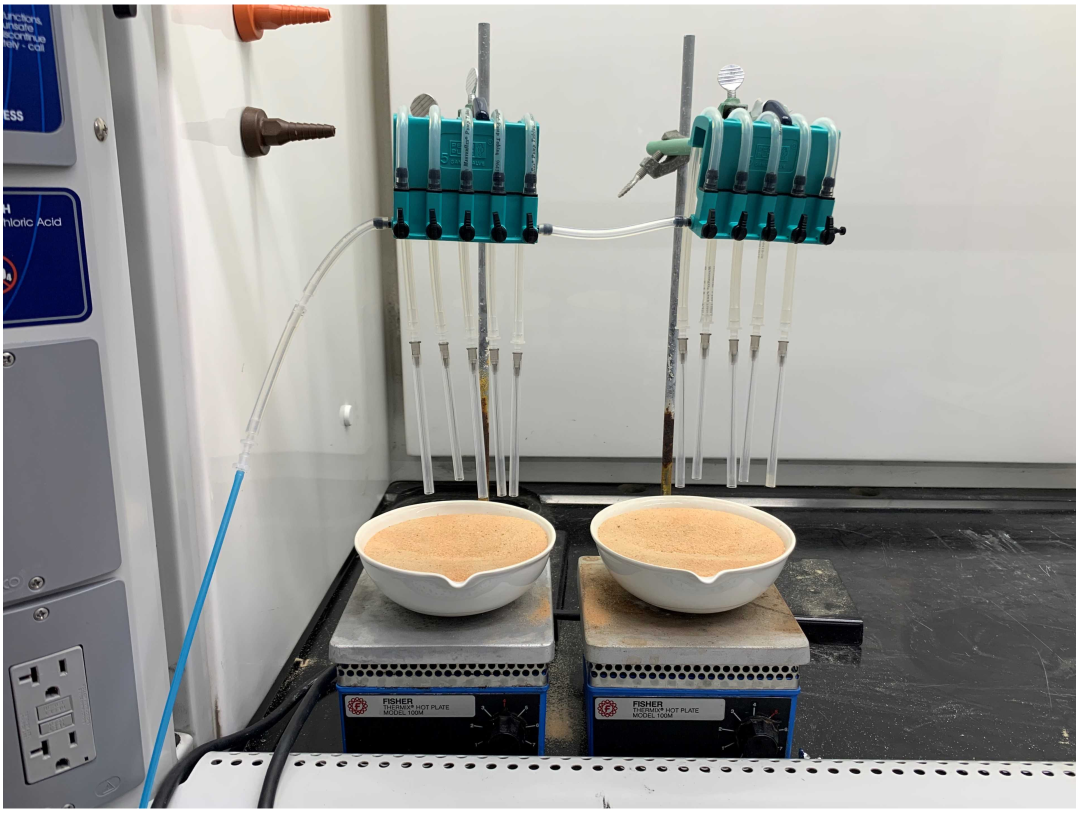

Image of a possible drying apparatus and sand baths (gas tank not pictured).

Set up the drying apparatus* and sand bath in a chemical hood via the following steps:

1. Suspend and secure drying apparatus using a clamp and ring stand
2. Use tubing and connectors to attach drying apparatus to gas tank[†]
3. Place sand bath on a hot plate beneath the drying apparatus

*Note that this drying apparatus is different from the one used to dry SPE cartridges in step 16.

[†]Either of the following gases may be used to dry nitric acid in ampules: high purity nitrogen or argon.

We use a combination of the following connectors, tubing, and other materials to construct the drying apparatus shown here:

- tubing: nylon tubing 4mm OD, 2.5mm ID (Shimadzu 016-43284-01), precision pump tubing L/S 16, 3.1mm (Masterflex ZY-96410-16)
- connectors: Male luer to male luer (Masterflex HV-30800-14), male luer lock to female luer (Masterflex ZY-45502-80), female luer to hosebarb 3/32" (Masterflex ZY-45501-02), Lok Tite Plastic 5 Gang Valve Aquarium Pump Accessory (PENN PLAX)
- other: sand (Fischer Brand S04286-8), evaporation dishes (CoorsTek 60204), ring stands (United Scientific Supplies S27848), ring stand clamps (Fischer Brand 05-769-7Q and 05-754Q), hotplates, hypodermic needles (Air-Tite™ N223)

Citation: Riley Barton, Sasha Wagner Measuring dissolved black carbon in water via aqueous, inorganic, high performance liquid chromatography of benzenepolycarboxylic acid (BPCA) molecular markers https://dx.doi.org/10.17504/protocols.io.5qpvoy2b9g4o/v2

This is an open access protocol distributed under the terms of the **Creative Commons Attribution License** (https://creativecommons.org/licenses/by/4.0/), which permits unrestricted use, distribution, and reproduction in any medium,

However, other materials and configurations may be used to construct a drying apparatus that works best for your lab setup. It is important to note that you are drying hot nitric acid in this step, so be sure to use materials that are compatible with acidic fumes.

40 Turn on hot plate and allow sand bath to heat up to approximately 60 °C . 1h

It usually takes approximately 1 hour for the bath to reach and stabilize at this temperature. You will need to determine the exact setting for your hot plate to reach this desired temperature.

41 Remove ampules from freezer and place in a rack. Gently flick the top of each ampule to dislodge any nitric acid solution that may be trapped near the sealed ampule tip and let the solution drip down to the bottom of the ampule.

42 Carefully break off the top of the ampules. Keep the ampule tops organized in a way that it is clear that each top corresponds to the bottom. At this point, the ampule tops still contain trace amounts of BPCA products and we will quantitatively transfer this material to the rest of the ampule in the next step.

Only open and prepare samples that you expect to dry down within the day. Keep other ampules sealed and stored in the freezer if you are unable to prepare them all. It takes ~1 hour for each batch of samples to dry down using the apparatus shown here.

Technique for breaking open ampules - Make sure there is no sample in the top/neck of ampule. If there is, then gently flick the ampule until all of the sample is in the bottom of the ampule. Hold the ampule bottom upright in one hand and, with your other hand, place your thumb under the bulb and your pointer finger on the opposite side up the neck. Break open the ampule away from you in one motion. If you struggle with opening ampules, you may choose to use an ampule-opening device.

Please see the online version of this protocol for a video of ampule-breaking technique

43 Quantitatively transfer BPCA oxidation products from ampule top to the rest of the solution in the ampule bottom.

43.1 Pipette 40 µL of concentrated nitric acid into the tops of each ampule.

Citation: Riley Barton, Sasha Wagner Measuring dissolved black carbon in water via aqueous, inorganic, high performance liquid chromatography of benzenepolycarboxylic acid (BPCA) molecular markers https://dx.doi.org/10.17504/protocols.io.5qpvoy2b9g4o/v2

This is an open access protocol distributed under the terms of the **Creative Commons Attribution License** (https://creativecommons.org/licenses/by/4.0/), which permits unrestricted use, distribution, and reproduction in any medium,

43.2 Roll and swirl the ampule top to rinse the entire inner surface area and to collect any BPCA-residue in the top of the ampule.

43.3 Using a fresh pipette tip, transfer the nitric acid rinsing from the ampule top to the corresponding bottom. Repeat this step for all samples, changing pipette tips between each sample to prevent cross contamination.

44 Evaporate the concentrated nitric acid contained in the ampules.

44.1 Gently push opened ampule into the sand to just below the shoulder of the ampule.

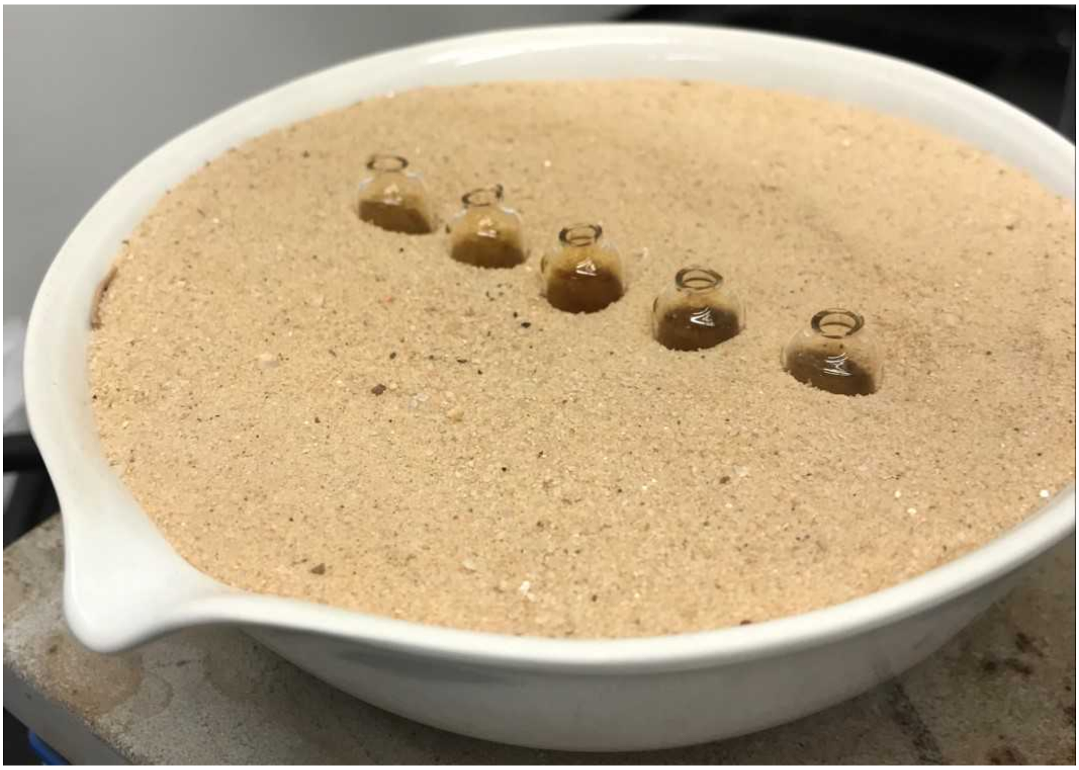

Image of ampules positioned in a sand bath.

Using the setup shown here, we can fit 5 ampules per sand bath. The exact configuration may vary depending upon how you build your drying apparatus.

Citation: Riley Barton, Sasha Wagner Measuring dissolved black carbon in water via aqueous, inorganic, high performance liquid chromatography of benzenepolycarboxylic acid (BPCA) molecular markers https://dx.doi.org/10.17504/protocols.io.5qpvoy2b9g4o/v2

This is an open access protocol distributed under the terms of the **Creative Commons Attribution License** (https://creativecommons.org/licenses/by/4.0/), which permits unrestricted use, distribution, and reproduction in any medium,

44.2 Remove steel needle covers and clean each needle with a Kimwipe soaked in methanol.

44.3 Insert needle into the headspace of the ampule so the tip of the needle is hovering just above the surface of the nitric acid in the ampule.

> **Take care when inserting and removing needles to and from the ampules** - There is a risk getting sand in your samples. If you accidentally introduce sand to the ampules, take notes on which samples were affected and check BPCA analyses later for possible signs of contamination. Since the BPCAs are chromatographically separated, contamination from sand is unlikely to interfere with DBC quantification results.

44.4 **SLOWLY** increase gas flow. The nitric acid solution in each ampule should shimmer slightly, not sputter aggressively. You may observe dark orange-to-light colored fumes emitting from the ampules.

> To optimize evaporation, you may adjust gas flow and/or needle height periodically during dry down as the liquid level decreases. This step requires a watchful eye and some degree of “babysitting” to ensure efficient evaporation.

44.5 After nitric acid has completely evaporated, turn off gas flow and remove needles from ampules and remove ampules from the sand bath. Gently wipe down the exterior of each ampule to remove excess sand.

> **Signs that nitric acid is completely evaporated** - If the steel needle and area around the opening of the ampule are completely dry, then it is likely that all nitric acid has been evaporated. If you remove the ampule and notice that some nitric acid remains, simply replace the ampule in the sand bath and continue drying.
>
> **Appearance of BPCA-containing residue** - The dry BPCA-containing residue left in the ampule is typically clear, white, or light yellow in color. The residue may not always be readily apparent to the naked eye.
>
> **Turn off gas flow BEFORE removing needles** - If you remove needles while gas is still flowing, then sand will blow all over the place.

Citation: Riley Barton, Sasha Wagner Measuring dissolved black carbon in water via aqueous, inorganic, high performance liquid chromatography of benzenepolycarboxylic acid (BPCA) molecular markers https://dx.doi.org/10.17504/protocols.io.5qpvoy2b9g4o/v2

This is an open access protocol distributed under the terms of the **Creative Commons Attribution License** (https://creativecommons.org/licenses/by/4.0/), which permits unrestricted use, distribution, and reproduction in any medium,

45 Repeat previous step until all samples are dried down. Wipe down needles with a Kimwipe soaked in methanol between each batch of samples added to sand bath to prevent cross contamination.

HPLC Analysis of BPCAs: Transfer of BPCAs from ampules to HPLC vials 30m

46 In this section, the BPCA-containing residue in the ampule is re-dissolved in mobile phase and transferred to the HPLC vial for analysis. 1h

Label HPLC vials with sample identifier or name, then add inserts to reduce interior volume.

Note that vial inserts may not be needed if mobile phase volume is sufficiently high (e.g., >0.5 mL). We recommend using inserts if you are following the exact protocol described here.

47

Transfer the target volume of mobile phase A to the ampule containing the dry BPCA-containing residue using a volumetric pipette. **Record the volume of mobile phase A added to the ampule.**

Mobile phase A is a dilute phosphoric acid solution (0.6 M; pH 1). See step 51 for additional details on formulation of HPLC mobile phases associated with this protocol.

If 0.5 mg of sample SPE-DOC is oxidized (see step 28), we typically recommend re-dissolving the BPCA-containing residue in 200 µL of mobile phase A for robust BPCA detection. However, mobile phase volumes may range from 150 to 250 µL depending on sample type and expected BPCA concentrations.

Recording the volume of mobile phase A added is necessary for determining accurate DBC concentrations in the original sample.

48 Rinse the entire interior surface of the ampule with the volume of mobile phase A added.

This step usually requires a combination of gentle shaking, rolling, and tilting of the ampule for a minute or two to effectively re-dissolve the BPCAs in the mobile phase A added. It is important to make sure that the mobile phase rinses every part of the interior surface of the ampule to ensure quantitative detection and analysis of BPCAs in a given sample. Whatever

Citation: Riley Barton, Sasha Wagner Measuring dissolved black carbon in water via aqueous, inorganic, high performance liquid chromatography of benzenepolycarboxylic acid (BPCA) molecular markers https://dx.doi.org/10.17504/protocols.io.5qpvoy2b9g4o/v2

This is an open access protocol distributed under the terms of the **Creative Commons Attribution License** (https://creativecommons.org/licenses/by/4.0/), which permits unrestricted use, distribution, and reproduction in any medium,

method you use to re-dissolve the BPCAs, keep your approach consistent among different samples. Take care not to spill any mobile phase during this step.

49 Using a clean glass or plastic pipette, transfer the mobile phase in the ampule, which now contains re-dissolved BPCAs, from the ampule to the insert in the corresponding HPLC vial.

When transferring the sample, place the pipette tip at the bottom of the vial insert and slowly deposit the sample to avoid bubble formation at the bottom of the insert.

Note that there may still be some mobile phase residue left in the ampule after transfer to the HPLC vial. This will not affect the accurate calculation of DBC concentrations because the concentration of BPCAs in the vial insert is representative of all BPCAs derived from the sample dissolved in the target volume of mobile phase A originally added to the ampule.

50

Cap the HPLC vials. At this point, samples can be analyzed immediately, or stored in the freezer for years without compromising sample integrity.

HPLC Analysis of BPCAs: Preparation of HPLC mobile phases and BPCA standard solutions 30m

51 Details regarding the HPLC analysis of BPCAs are previously described by Wagner et al. (2017). 5m

Prepare mobile phase A = [M]**0.6 Molarity (M)** phosphoric acid (pH 1).

Add ~500 mL of ultrapure water to a clean 1 L volumetric flask, add 40 mL of o-phosphoric acid (14.6 M), then fill flask with ultrapure water and mix until homogeneous.

Wagner S, Brandes J, Goranov AI, Drake TW, Spencer RGM, Stubbins A. (2017). Online quantification and compound-specific stable isotopic analysis of black carbon in environmental matrices via liquid chromatography-isotope ratio mass spectrometry. Limnol. Oceanogr.: Meth.
https://doi.org/10.1002/lom3.10219

Citation: Riley Barton, Sasha Wagner Measuring dissolved black carbon in water via aqueous, inorganic, high performance liquid chromatography of benzenepolycarboxylic acid (BPCA) molecular markers https://dx.doi.org/10.17504/protocols.io.5qpvoy2b9g4o/v2

This is an open access protocol distributed under the terms of the **Creative Commons Attribution License** (https://creativecommons.org/licenses/by/4.0/), which permits unrestricted use, distribution, and reproduction in any medium,

52 Prepare mobile phase B = [M]**20 millimolar (mM)** sodium phosphate buffer (pH 6). 5m

Add 2.42 g of NaH_2PO_4 monohydrate and 0.64 g of Na_2HPO_4 heptahydrate to a 1 L volumetric flask, then fill flask with ultrapure water and mix until salts are completely dissolved.

53 Prepare stock solution of BPCA standards = [M]**5000 micromolar (µM)** of BPCA-carbon (BPCA-C). 15m

Stock solution concentrations used here are consistent with those described by Wagner et al. (2017). Note that concentrations are µM of BPCA-C (i.e. carbon concentrations), not BPCA molecular concentrations - this is taken into account during quantification of DBC via the conversion factor described in step 66.

Stock solution can be stored in the refrigerator for 6 months. However, peak areas of standard stock solution should be monitored with each run to examine for degradation. Keep in mind that conditions such as column aging and changes in temperature can alter standard peak areas.

All BPCAs are included in the standard solution but not all of them are measured for dissolved black carbon concentrations. Please see Wagner et al. (2017) and Stubbins et al. (2015) supporting information for further details and justification.

53.1 Using a microbalance, weigh out each BPCA standard to the target mass listed in the table below. Transfer all measured BPCAs to the same 100 mL volumetric flask.

Since it is difficult to weigh out accurately to the µg, **record the actual mass of each BPCA standard that was weighed out.** Actual masses will be used to calculate actual BPCA stock solution concentrations.

Citation: Riley Barton, Sasha Wagner Measuring dissolved black carbon in water via aqueous, inorganic, high performance liquid chromatography of benzenepolycarboxylic acid (BPCA) molecular markers https://dx.doi.org/10.17504/protocols.io.5qpvoy2b9g4o/v2

This is an open access protocol distributed under the terms of the **Creative Commons Attribution License** (https://creativecommons.org/licenses/by/4.0/), which permits unrestricted use, distribution, and reproduction in any medium,

A	B	C
BPCA Standard	*Target Mass (mg)*	*Actual Mass (mg)*
1,2,3-benzenetricarboxylic acid	11.680	11.661
1,2,4-benzenetricarboxylic acid	11.680	11.638
1,3,5-benzenetricarboxylic acid	11.680	11.655
1,2,4,5-benzenetetracarboxylic acid	12.700	12.749
benzenepentacarboxylic acid (B5CA)	13.560	13.618
benzenehexacarboxylic acid (B6CA)	14.260	14.145

Table showing target masses for each BPCA standard and an example of actual masses measured for each BPCA standard.

Weighing tip - Cut weigh paper into small squares and fold in half diagonally to create a crease before weighing out the BPCA standard. This crease will make it easier to transfer the standard material to the volumetric flask.

Why does the standard mixture contain additional BPCA standards (besides B5CA and B6CA)? Although benzenetri- and benzenetetra-carboxylic acids are not used to quantify DBC as part of this protocol, we continue to incorporate these standards into our analyses to assess sample peak separation and to evaluate overall chromatographic performance.

53.2 After all BPCA standards have been weighed out and transferred to the volumetric flask, fill with mobile phase A and mix to dissolve BPCAs.

Some BPCAs are less soluble than others, therefore you may want to use a warm sonic bath to encourage dissolution.

54 Conduct a series of simple dilutions to produce solutions of 2000, 1000, 500, 200, 100, 50, and 25 uM BPCA-C standards. These solutions will be used for external calibration of BPCAs in unknown samples.

We typically use 10 mL volumetric flasks to dilute the BPCA stock solution.

55 Transfer ~1 mL of mobile phase A directly into a clean HPLC vial. Label as "blank" and cap and set aside.

Citation: Riley Barton, Sasha Wagner Measuring dissolved black carbon in water via aqueous, inorganic, high performance liquid chromatography of benzenepolycarboxylic acid (BPCA) molecular markers https://dx.doi.org/10.17504/protocols.io.5qpvoy2b9g4o/v2

This is an open access protocol distributed under the terms of the **Creative Commons Attribution License** (https://creativecommons.org/licenses/by/4.0/), which permits unrestricted use, distribution, and reproduction in any medium,

56 Transfer ~1 mL of each BPCA standard solution directly into a clean, separate HPLC vials. Label each vial with the BPCA standard concentration and cap and set aside.

HPLC Analysis of BPCAs: Chromatographic separation of individual BPCAs 2d

57 The HPLC separation method described here was first presented by Wagner et al. (2017) for online compound-specific stable carbon isotopic analysis. In this protocol, we use the same chromatographic parameters, but with UV detection, for the efficient quantification of DBC.

HPLC system and user-related assumptions - For the purposes of this protocol, we assume that the user will have access to an HPLC system that is equipped with an autosampler, binary or quaternary pump (for gradient separation), a UV or photo diode array detector, and software to assemble methods and sequences and for instrumental operation. We also assume that the user has basic knowledge of how to operate an HPLC system and/or has access to someone who can assist them with basic operation of an HPLC system. For example, we do not go into detail about how to flush solvent lines or properly hook up and equilibrate an HPLC column.

HPLC instrument settings for the chromatographic separation of BPCAs are as follows:

- Column: Agilent Poroshell 120 phenyl-hexyl column (4.6 x 150 mm, 2.7 µm) with corresponding guard column (4.6 x 5 mm, 2.7 µm)
- Mobile phase A: Phosphoric acid (0.6 M; pH 1)
- Mobile phase B: Sodium phosphate buffer (20 mM; pH 6)
- Flow rate: 500 µL min^{-1}
- Column temperature: 25°C (if available)
- Autosampler temperature: 25°C (if available)
- Injection volume: 25 µL
- Peak detection: 240 nm

Set up the HPLC according to the parameters listed above.

58 Using the HPLC software, create a method file for gradient separation of BPCAs.

A	B	C
Time (min)	Mobile phase A (vol. %)	Mobile phase B (vol. %)
0	70	30
15	0	100
35	0	100
35.01	70	30
45	70	30

Table showing optimized gradient elution of BPCAs from Wagner et al. (2017).

Citation: Riley Barton, Sasha Wagner Measuring dissolved black carbon in water via aqueous, inorganic, high performance liquid chromatography of benzenepolycarboxylic acid (BPCA) molecular markers https://dx.doi.org/10.17504/protocols.io.5qpvoy2b9g4o/v2

This is an open access protocol distributed under the terms of the **Creative Commons Attribution License** (https://creativecommons.org/licenses/by/4.0/), which permits unrestricted use, distribution, and reproduction in any medium,

Wagner S, Brandes J, Goranov AI, Drake TW, Spencer RGM, Stubbins A. (2017). Online quantification and compound-specific stable isotopic analysis of black carbon in environmental matrices via liquid chromatography-isotope ratio mass spectrometry. Limnol. Oceanogr.: Meth..
https://doi.org/10.1002/lom3.10219

59 Order your HPLC sequence by first starting with a blank injection, then BPCA standards in order of decreasing concentration, then another blank, and then the unknown samples. Perform a blank injection and re-run one of the mid-concentration BPCA standards every ten or so samples - Doing so enables you to assess the effects of potential carryover (uncommon) and instrumental/column drift (more common).

Citation: Riley Barton, Sasha Wagner Measuring dissolved black carbon in water via aqueous, inorganic, high performance liquid chromatography of benzenepolycarboxylic acid (BPCA) molecular markers https://dx.doi.org/10.17504/protocols.io.5qpvoy2b9g4o/v2

This is an open access protocol distributed under the terms of the **Creative Commons Attribution License** (https://creativecommons.org/licenses/by/4.0/), which permits unrestricted use, distribution, and reproduction in any medium,

A	B
Sample Type	Sample ID
blank	blank
BPCA standard	5000 µM
BPCA standard	2000 µM
BPCA standard	1000 µM
BPCA standard	500 µM
BPCA standard	200 µM
BPCA standard	100 µM
BPCA standard	50 µM
BPCA standard	25 µM
blank	blank
sample	river1
sample	river2
sample	river3
sample	river4
sample	river5
sample	ocean1
sample	ocean2
sample	ocean3
sample	ocean4
sample	ocean5
blank	blank
BPCA standard	1000 µM
sample	lake1
...	...

Table showing suggested HPLC sequence setup. Note that the sample IDs in column B are fictional.

60 Place blank, BPCA standard, and sample vials into the appropriate autosampler positions.

> If you are analyzing samples that were previously stored in the freezer, shake the vial downward in a flicking motion to ensure the liquid in the vial settles to the bottom of the insert and the solution is re-mixed. This ensures that there is not a concentration gradient nor bubbles in the vial due to freezing/thawing. You may also use a pipette to mix and deposit your sample, but this may introduce extraneous material to the vial.

61 At this point, the HPLC system should be set up for BPCA analysis and the column is properly equilibrated with mobile phase (70% A and 30% B).

Citation: Riley Barton, Sasha Wagner Measuring dissolved black carbon in water via aqueous, inorganic, high performance liquid chromatography of benzenepolycarboxylic acid (BPCA) molecular markers https://dx.doi.org/10.17504/protocols.io.5qpvoy2b9g4o/v2

This is an open access protocol distributed under the terms of the **Creative Commons Attribution License** (https://creativecommons.org/licenses/by/4.0/), which permits unrestricted use, distribution, and reproduction in any medium,

Run the HPLC sequence.

Note that the mobile phase B reservoir will empty faster than the mobile phase A reservoir. Depending upon the length of the sequence, it could take several days to analyze a full sample set. We suggest checking on the instrument at least daily to refill mobile phase reservoirs and to check for leaks or other observable issues with separation. We also suggest checking whether the BPCA standards ran well before continuing to analyze the rest of the dataset.

62 Examine the chromatogram for each blank run. 45m

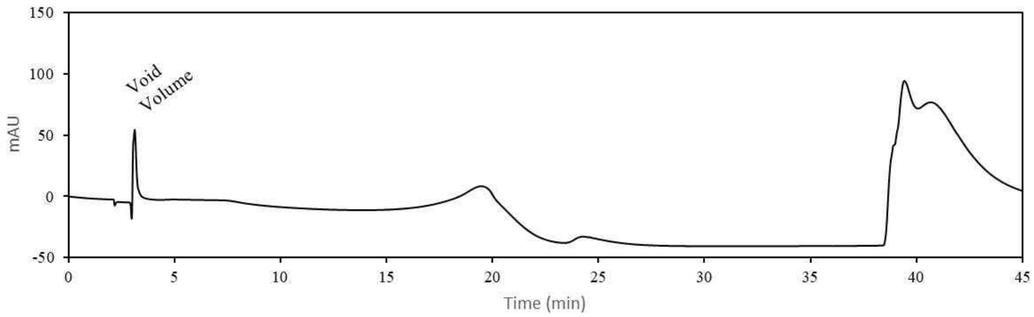

Chromatogram of a blank sample (injection of 0.6M phosphoric acid).

There should be no observable peaks except the void volume around a retention time of ~3 minutes. Note that there is some degree of baseline drift due to the mobile phase gradient.

63 Examine the chromatogram for each BPCA standard run. 45m

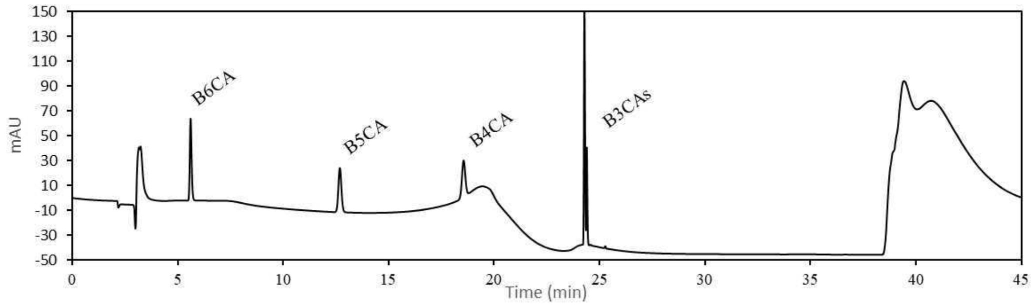

Chromatogram of a standard mixture

In each BPCA standard chromatogram, you should observe baseline separation of B6CA at a retention time of ~5 minutes and baseline separation of B5CA at a retention time of ~12

Citation: Riley Barton, Sasha Wagner Measuring dissolved black carbon in water via aqueous, inorganic, high performance liquid chromatography of benzenepolycarboxylic acid (BPCA) molecular markers https://dx.doi.org/10.17504/protocols.io.5qpvoy2b9g4o/v2

This is an open access protocol distributed under the terms of the **Creative Commons Attribution License** (https://creativecommons.org/licenses/by/4.0/), which permits unrestricted use, distribution, and reproduction in any medium,

minutes. The 1,2,4,5-benzenetetracarboxylic acid standard is baseline separated at a retention time of ~18 minutes and the benzenetricarboxylic acids usually co-elute around a retention time of ~24 minutes.

64 Examine the chromatogram for each sample run. 45m

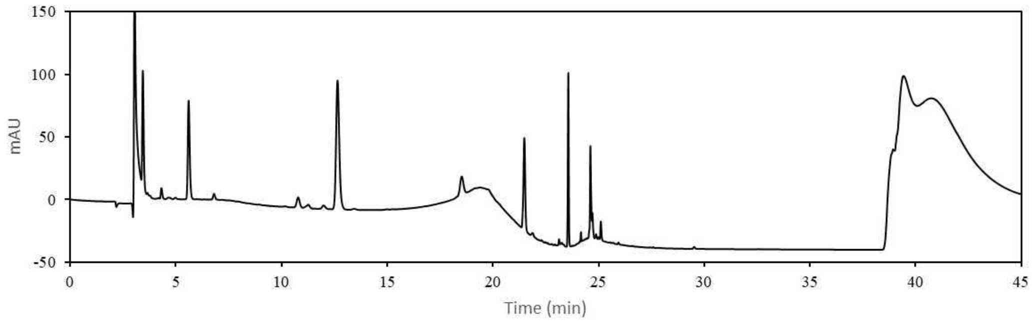

Chromatogram of dissolved organic matter sample

Sample oxidation products contain target BPCAs in addition to numerous other organic components. Compounds B6CA and B5CA are usually baseline-separated and are identified by comparison of the absorbance spectrum (preferred, if available) and/or sample peak retention times with that of BPCA standard runs.

65 To our current knowledge, sample-containing HPLC vials can be stored in the freezer indefinitely.

BPCAs stored in mobile phase A are stable and can be reanalyzed at a future time if desired, or used for online compound-specific stable carbon isotopic analysis of BPCAs following Wagner et al. (2017).

Calculation of DBC Concentrations 1h

66 Using an external calibration approach, establish calibration equations for B6CA and B5CA.

Using the HPLC software, integrate the area under B6CA and B5CA peaks for each BPCA standard run. Enter peak retention times and peak areas for B6CA and B5CA into the spreadsheet below (DBC Calculations tab). Also enter the mass of B6CA and B5CA weighed out for the 5000 µM-C BPCA stock solution. If these steps are followed, the standard concentrations will be automatically converted from µM-C to µM-BPCA.

The slope and intercept of the B6CA and B5CA will be calculated automatically and carried through to the DBC calculations spreadsheet.

Example PPL-BPCA Calculations.xlsx

Citation: Riley Barton, Sasha Wagner Measuring dissolved black carbon in water via aqueous, inorganic, high performance liquid chromatography of benzenepolycarboxylic acid (BPCA) molecular markers https://dx.doi.org/10.17504/protocols.io.5qpvoy2b9g4o/v2

This is an open access protocol distributed under the terms of the **Creative Commons Attribution License** (https://creativecommons.org/licenses/by/4.0/), which permits unrestricted use, distribution, and reproduction in any medium,

The approach used to integrate BPCA peak areas should be consistent across all samples analyzed in a particular dataset.

We recommend plotting a graph of the linear relationship between peak area and concentration for B6CA and B5CA before moving on to the next step to ensure that calibration equations are reliable for quantification of BPCAs in unknown samples.

67 Using the HPLC software, integrate the area under B6CA and B5CA peaks for each sample run and BPCA standard runs that are interspersed within the sample queue.

Enter peak retention times and peak areas for B6CA and B5CA into the calculations spreadsheet.

68 The data entered into the spreadsheet up to this point is used to automatically calculate DBC concentrations in the original sample. We strongly encourage users to try these calculations by hand to fully understand how the final DBC concentrations are obtained.

The equations used in the spreadsheet are summarized below.

Calculations

$$\boldsymbol{[BPCA]_{in\ HPLC\ vial}}$$

$$[BPCA]_{in\ HPLC\ vial} = Peak\ Area_{BPCA} * Slope_{\ [BPCA]\ v.\ Peak\ Area} + Intercept_{[BPCA]\ v.\ Peak\ Area}$$

(units: uM = Area(uM/Area) + uM)*

$$\boldsymbol{[BPCA]_{in\ MeOH\ eluate}}$$

$$[BPCA]_{in\ MeOH\ eluate} = [BPCA]_{in\ HPLC\ vial} * V_{mobile} \div V_{MeOH\ dried}$$

*(units: uM = uM*mL/mL)*

$$\boldsymbol{[BPCA]_{in\ sample}}$$

$$[BPCA]_{in\ sample} = [BPCA]_{in\ MeOH\ eluate} * V_{MeOH\ through\ PPL} \div V_{sample}$$

(units: uM = uM*mL/mL)

$$\boldsymbol{[B6CA + B5CA]_{(nM)}}$$

$$[B6CA + B5CA]_{(nM)} = ([B6CA]_{(uM)} + [B5CA]_{(uM)}) * 1000$$

(units: nM = (uM+uM)*1000

$$\boldsymbol{[DBC]_{(uM)}}$$

$$[DBC]_{(uM)} = 0.0891 * [B6CA + B5CA]_{(nM)}^{0.9175}$$

Image of calculations used to calculate the concentration of DBC in the original sample from individual BPCA peak areas.

Note that we use the Stubbins et al. (2015) conversion factor for quantification of DBC

Citation: Riley Barton, Sasha Wagner Measuring dissolved black carbon in water via aqueous, inorganic, high performance liquid chromatography of benzenepolycarboxylic acid (BPCA) molecular markers https://dx.doi.org/10.17504/protocols.io.5qpvoy2b9g4o/v2

This is an open access protocol distributed under the terms of the **Creative Commons Attribution License** (https://creativecommons.org/licenses/by/4.0/), which permits unrestricted use, distribution, and reproduction in any medium,

concentrations from B6CA and B5CA concentrations.

The spreadsheet also contains calculations of B6CA:B5CA and DBC:DOC ratios, which are a measure of the relative degree of condensed aromaticity of DBC measured and the relative proportion of DBC in bulk DOC. See main text of this protocol or Wagner et al. (2017) for more information on these ratios.

When publishing data obtained using this protocol, we ask users to include "in sample" concentrations for B6CA, B5CA, and DBC.

Stubbins A, Spencer RGM, Mann PJ, Holmes RM, McClelland JW, Niggemann J, Dittmar T. (2015). Utilizing colored dissolved organic matter to derive dissolved black carbon export by arctic rivers. Front. Earth Sci..
https://doi.org/10.3389/feart.2015.00063

Citation: Riley Barton, Sasha Wagner Measuring dissolved black carbon in water via aqueous, inorganic, high performance liquid chromatography of benzenepolycarboxylic acid (BPCA) molecular markers https://dx.doi.org/10.17504/protocols.io.5qpvoy2b9g4o/v2

This is an open access protocol distributed under the terms of the **Creative Commons Attribution License** (https://creativecommons.org/licenses/by/4.0/), which permits unrestricted use, distribution, and reproduction in any medium,
